# Supplementary material for: Aiolos represses CD4+ T cell cytotoxic programming via reciprocal regulation of TFH transcription factors and IL-2 sensitivity
Source: Nat Commun. 2023 Mar 24;14:1652. doi: 10.1038/s41467-023-37420-0 (PMC10039023; doi:10.1038/s41467-023-37420-0)

Supplementary Table 1. Primers used for qRT-PCR

Gene (murine)	Forward (5' to 3')	Reverse (5' to 3')
Ikzf3	CCGACTGTGGAGCTGAAAAGC	CCTGCATCTTCGTCTTCATTGG
Bcl6	CCAACCTGAAGACCCACACTC	GCGGACATGGCTCTTCAGAGTC
Tbx21	GTGACTGCCTACCAGAACGC	AGGGGACACTCGTATCAACAG
Gata3	CCGCAGGCATTGCAAAGGTA	CCAAGGCACGATCCAGCACA
Tcf7	TCCTGCGGATATAGACAGCACTTCC	ATATGGCTGCAGCTCCTGCTTC
Zfp831	CGCCTGAGCTTCCCTATCCATCAG	GCTCTCTGCCAGAAACTCCCTTC
Ifng	CTACCTTCTTCAGCAACAGC	GCTCATTGAATGCTTGGCGC
Gzmb	TCCTCCTGCTACTGCTGACCTTG	CACATATGCCCTCAGGCTGCTG
Eomes	AGAGGACGGTGTGGAGGACTTG	AACGCCGTACCGACCTCCAG
Prf1	GCTGAGAAGACCTATCAGGACCAG	GCCTGTGGTAAGCATGCTCTGTG
Il2ra	CCACAACAGACATGCAGAAGCC	GCAGGACCTCTCTGTAGAGCCTTG
Il2rb	GGCCATGGCTGAAGACAGTTCTC	CGGCCTTGGAATCTCCGTCGAG
Prdm1	CTTGTGTGGTATTGTCGGGAC	CACGCTGTACTCTCTCTTGG

Supplementary Table 2. Primers used for ChIP qPCR

Gene	Forward (5' to 3')	Reverse (5' to 3')
Bcl6 Ctrl.	GTACTCCAACAACAGCACAGC	GTGGCTCGTTAAATCACAGAG
Bcl6 Prom.	CGAGTTTATGGGTAGGAGAGG	GTCTTCGCTGTAGCAAAGCTCG
Zfp831 Ctrl.	GTGTTAACATGCTGGTCTCCCC	CGATGCTCTCCTCATTGACC
Zfp831 Enhc.	CTGTTGATGAAAACACTCGCGC	GGGAGGGAACACATGCTTCC
Il2ra Ctrl.	GCTCATGGCCTGCTTAACAG	CTCTCTGTCTCTGTCTCTCTC
Il2ra Prom.	GGACTCATAAGTGAAGCCTG	GCCTACTCTGTTCTGTGATCTC
Prdm1 Ctrl.	GTCACCACTCAACTTCAGACCAGAG	GCAGTATCCAGGACAACTTTCTGCTG
Prdm1 Enhc.	GCCTCTGTACTTGTGTTTCCTACACC	GGCAGGGTTCCAAGTATTCATCTG
Eomes Prom.	CACGATCTGACAGTTACACTAATGC	CACTTTCATCCGATTTCTGACAC
Ifng Prom.	GTGCTGTGCTCTGTGGATGAG	CCTTTCGACTCCTTGGGCTC
Gzmb Prom.	GCCTGATGACGTCTTCTGAG	GGGACTCTGATACCATAGGCTAC
Prf1 Prom.	GGACAGGAAGTGGGTACCAGCTTTG	GCCGCTTCACATCTCAGCTCC

Supplementary Figure 1

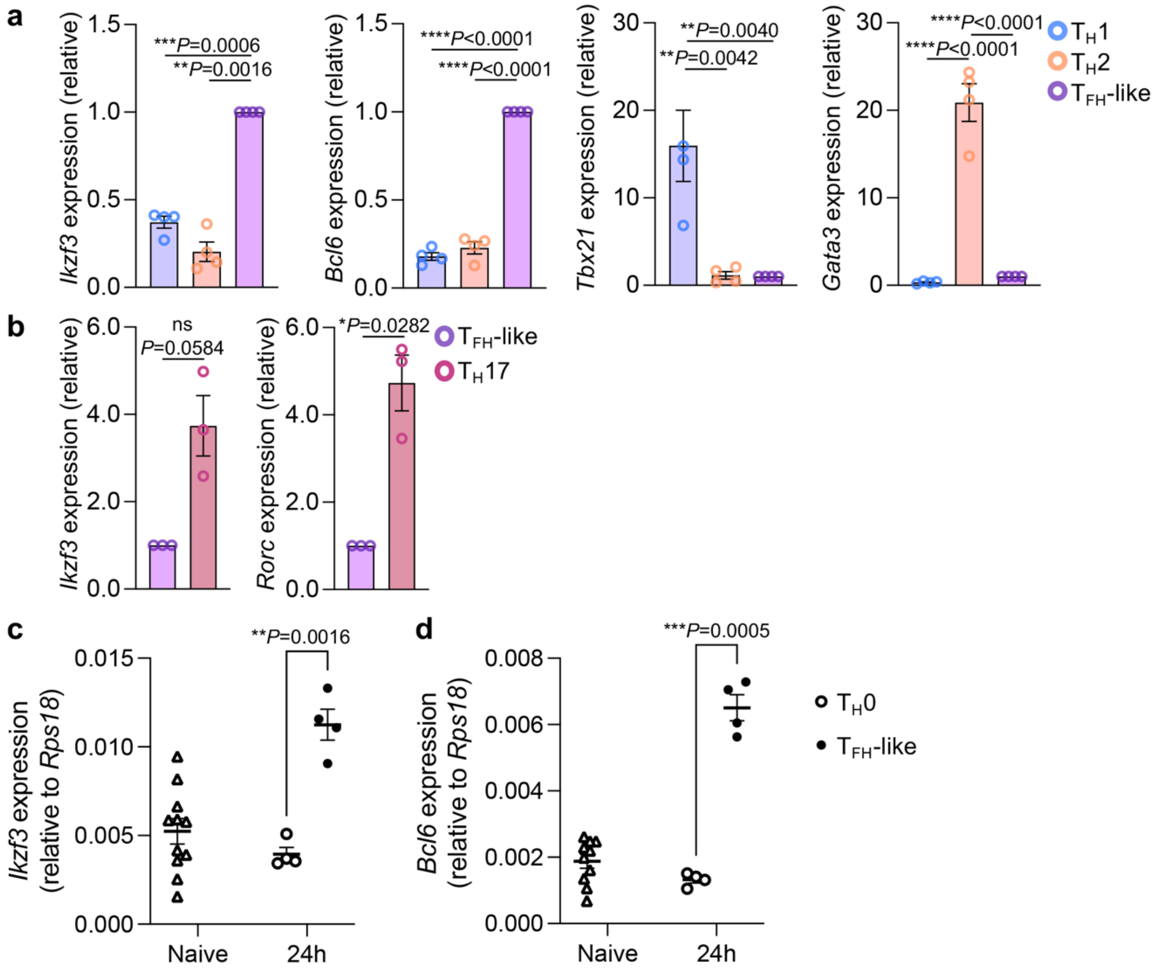

Supplementary Figure 1. Aiolos transcript expression is elevated in T_{FH}-like cells and correlates with that of *Bcl6*. (**a-b**) Analysis of Aiolos transcript expression across T helper cell populations. Naïve murine $CD4^+$ T cells were cultured under (**a**) T_H1, T_H2, and T_{FH}, or (**b**) T_{FH} and T_H17-polarizing conditions for 3 days. RNA was isolated and transcript analysis for the indicated genes was performed via qRT-PCR. For a, data are normalized and presented relative to the *Rps18* control. For b, data were normalized to the *Rps18* control and presented relative to the T_{FH} sample (For 'a' n = 4, for 'b' n=3. Data are presented as mean ± s.e.m; $^*P < 0.05$, $^{**}P < 0.01$, $^{***}P < 0.001$, $^{****}P < 0.0001$; one-way ANOVA with Tukey's multiple comparisons (a); two-sided, paired Student's t-test (b)). (**c-d**) qRT-PCR analysis of Aiolos (*Ikzf3*) and *Bcl6* expression in WT naïve $CD4^+$ T cells and T_H0 (non-polarizing control) or T_{FH}-polarized $CD4^+$ T cells cultured for 24h. Data are normalized and presented as expression relative to *Rps18* control. Data are compiled from 2 independent experiments, and each point represents a separate biological replicate. (n=4 ± s.e.m; $^{**}P < 0.01$, $^{***}P<0.001$; two-sided, paired Student's *t*-test). Source data are provided as a Source Data file.

Supplementary Figure 2

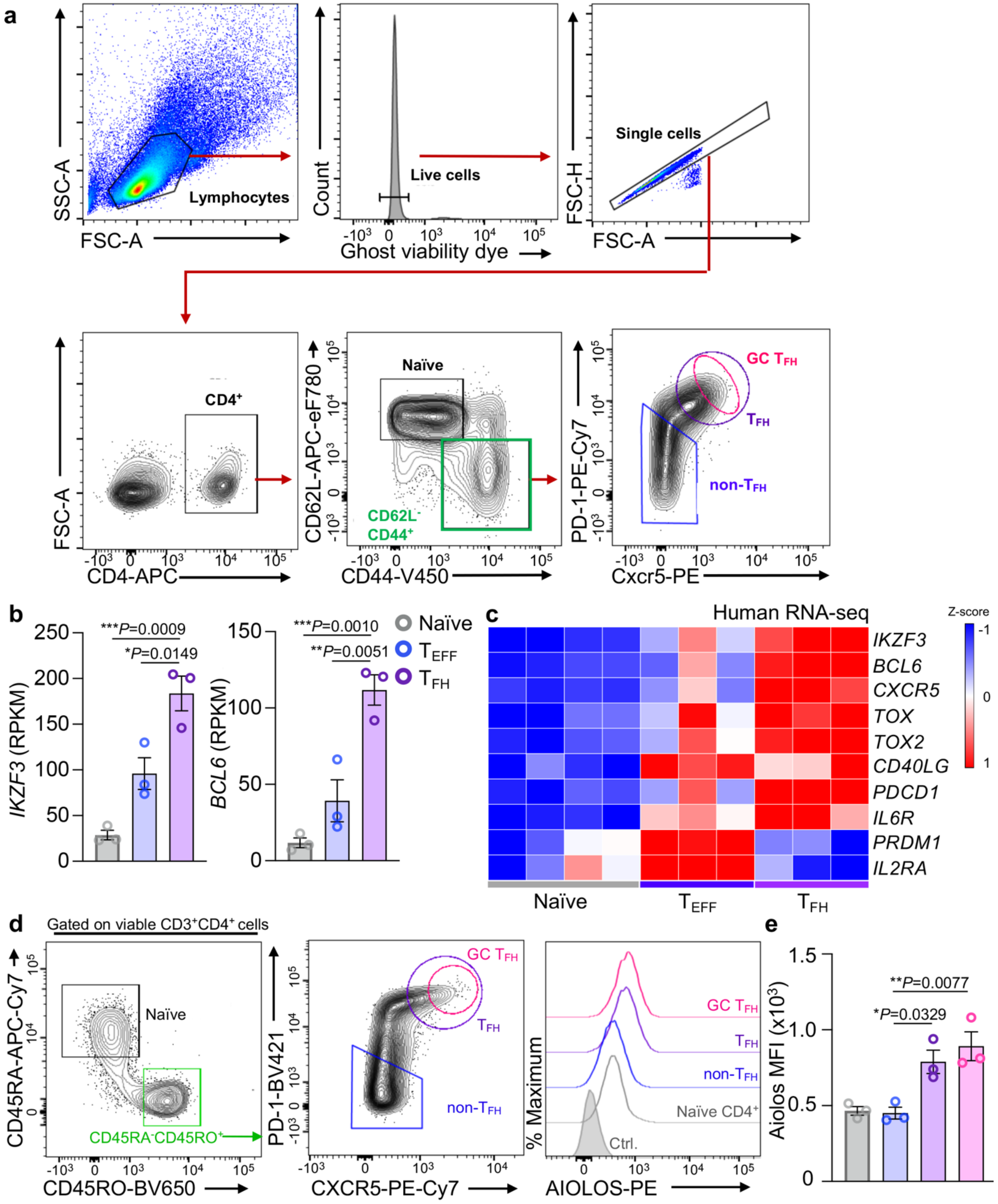

Supplementary Figure 2. Aiolos protein expression is elevated in both human and murine T_{FH} cell populations *in vivo*. (**a**) Representative gating strategy utilized for analysis of $CD4^{+}$ T cell populations generated during influenza infection. Example gating for T_{FH} populations is shown. **(b-c)** Publicly available RNA-seq data (GEO# GSE58596 [https://www.ncbi.nlm.nih.gov/geo/query/acc.cgi?acc=GSE58596]) from human tonsillar $CD4^{+}$ naïve, effector (T_{EFF}), and T_{FH} populations. Data are presented as reads per kilobase of transcript per million mapped reads (RPKM) (**b**) or as a heatmap with reads normalized by row (gene) (**c**). Data are compiled from 3 biological replicates representing 3 individual donors (n = 3 ± s.e.m; *P < 0.05, **P < 0.01, ***P <0.001; one-way ANOVA with Tukey's multiple comparison test). **(d-e)** Representative flow cytometry data of AIOLOS expression in human $CD4^{+}$ T cell populations isolated from pediatric tonsils. Median fluorescence intensity was quantitated for naïve ($CD3^{+}CD4^{+}CD8^{-}CD45RA^{+}CD45RO^{-}$; grey bar), non-$T_{FH}$ effector ($CD3^{+}CD4^{+}CD8^{-}CD45RA^{-}$ CD45RO+PD-$1^{lo}CXCR5^{-}$; blue bar), T_{FH} ($CD3^{+}CD4^{+}CD8^{-}CD45RA^{-}CD45RO^{+}$PD-$1^{hi}CXCR5^{+}$; purple bar), and germinal center (GC) T_{FH} ($CD3^{+}CD4^{+}CD8^{-}CD45RA^{-}CD45RO^{+}$PD-$1^{hi}$/$CXCR5^{hi}$; pink bar) populations. Data are compiled from two independent experiments and each point represents an individual donor (n = 3 ± s.e.m; *P < 0.05, **P < 0.01; one-way ANOVA with Tukey's multiple comparison test). Source data are provided as a Source Data file.

Supplementary Figure 3

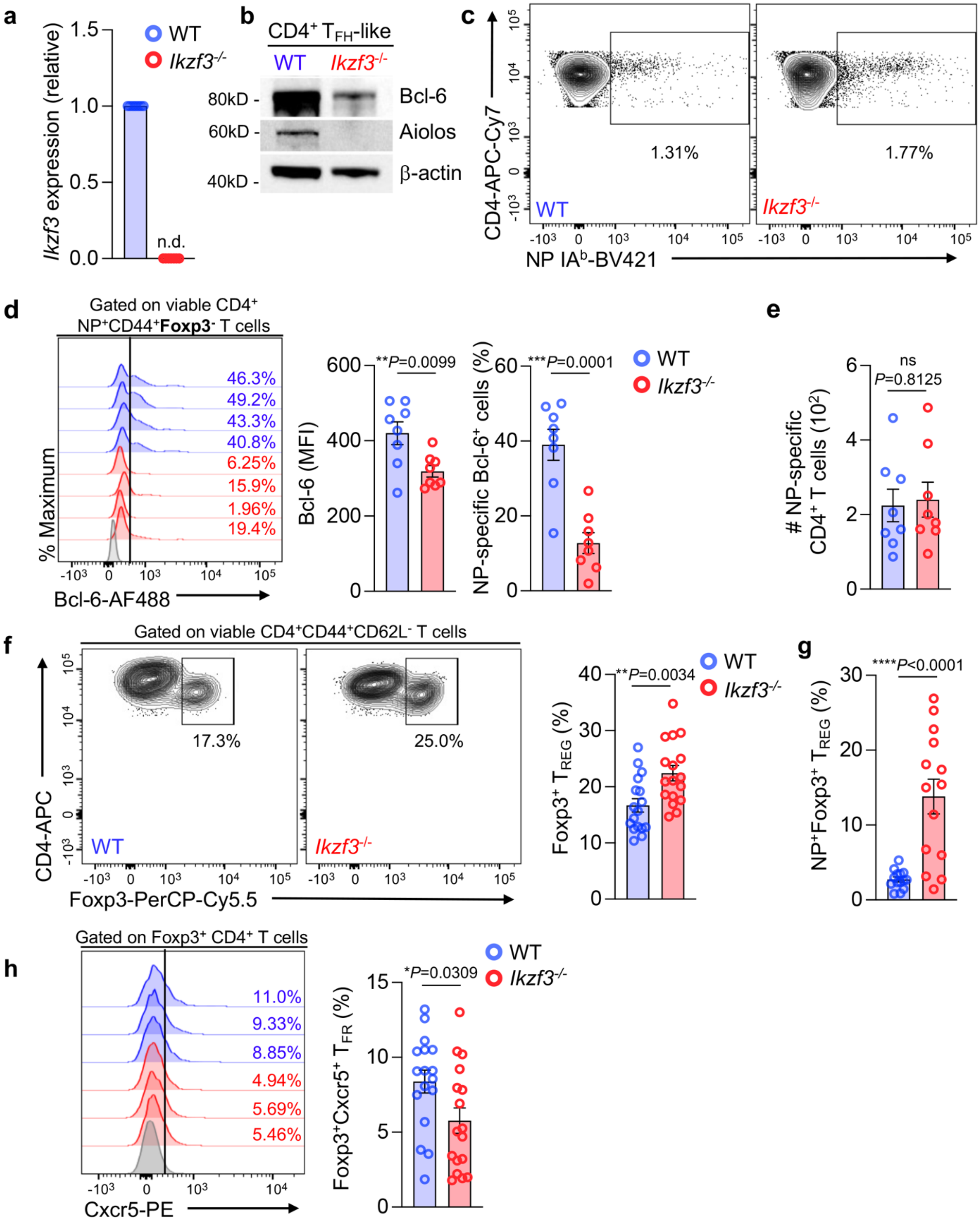

Supplementary Figure 3. Aiolos deficiency results in disrupted Bcl-6^{+} and T_{FR} populations, and augmented T_{REG} populations, during influenza virus infection. (**a-b**) Naïve WT or Aiolos-deficient (*Ikzf3*$^{-/-}$) CD4^{+} T cells were cultured under T_{FH}-polarizing conditions for 3 days. (**a**) Analysis of Aiolos (*Ikzf3*) expression via qRT-PCR (n=7 ± s.e.m; n.d., not detected). Data were normalized to *Rps18* and presented relative to the WT sample. Data are compiled from 6 independent experiments. (**b**) Analysis of the indicated proteins was performed via immunoblot. b-actin serves as a loading control. Image is representative from 2 biological replicates from 2 independent experiments. (**c-g**) Naïve WT or *Ikzf3*$^{-/-}$ C57BL/6 mice were infected intranasally with 30 PFU influenza (A/PR8/34; "PR8"). (**c**) Influenza nucleoprotein-specific CD4^{+} T cells were identified using pMHCII tetramers. Representative gates are shown for WT and *Ikzf3*$^{-/-}$ samples. (**d**) Bcl-6 protein expression was assessed in viable antigen-specific CD4^{+} T cells after 6 days of infection. Median fluorescence intensity (MFI) and percent antigen-specific Bcl6^{+} cells are shown. Data presented are from two independent experiments (n=8 ± s.e.m; **P < 0.01, ***P < 0.01; two-sided, unpaired Student's t-test). (**e**) Enumeration of influenza nucleoprotein (NP)-specific CD4^{+} T cells after 6 days of infection (n=8 ± s.e.m; two-sided, unpaired Student's t-test). (**f-g**) Foxp3^{+} T_{REG} frequency was assessed in WT or *Ikzf3*$^{-/-}$ mice 8 days post-infection in viable bulk (**f**) or antigen-specific (**g**) CD4^{+} T cell populations. Representative gating is provided in f. Data are compiled from 4 independent experiments (n=17 ± s.e.m; **P < 0.01, ****P < 0.0001; two-sided, unpaired Student's t-test). (**h**) Foxp3^{+}Cxcr5^{+} T_{FR} frequency was assessed in WT or *Ikzf3*$^{-/-}$ mice 8 days post-infection in viable CD4^{+} T cell populations. Representative gating is provided in h. Data are compiled from 4 independent experiments (n=17 ± s.e.m; **P < 0.01, ****P < 0.0001; two-sided, unpaired Student's t-test). Source data are provided as a Source Data file.

Supplementary Figure 4

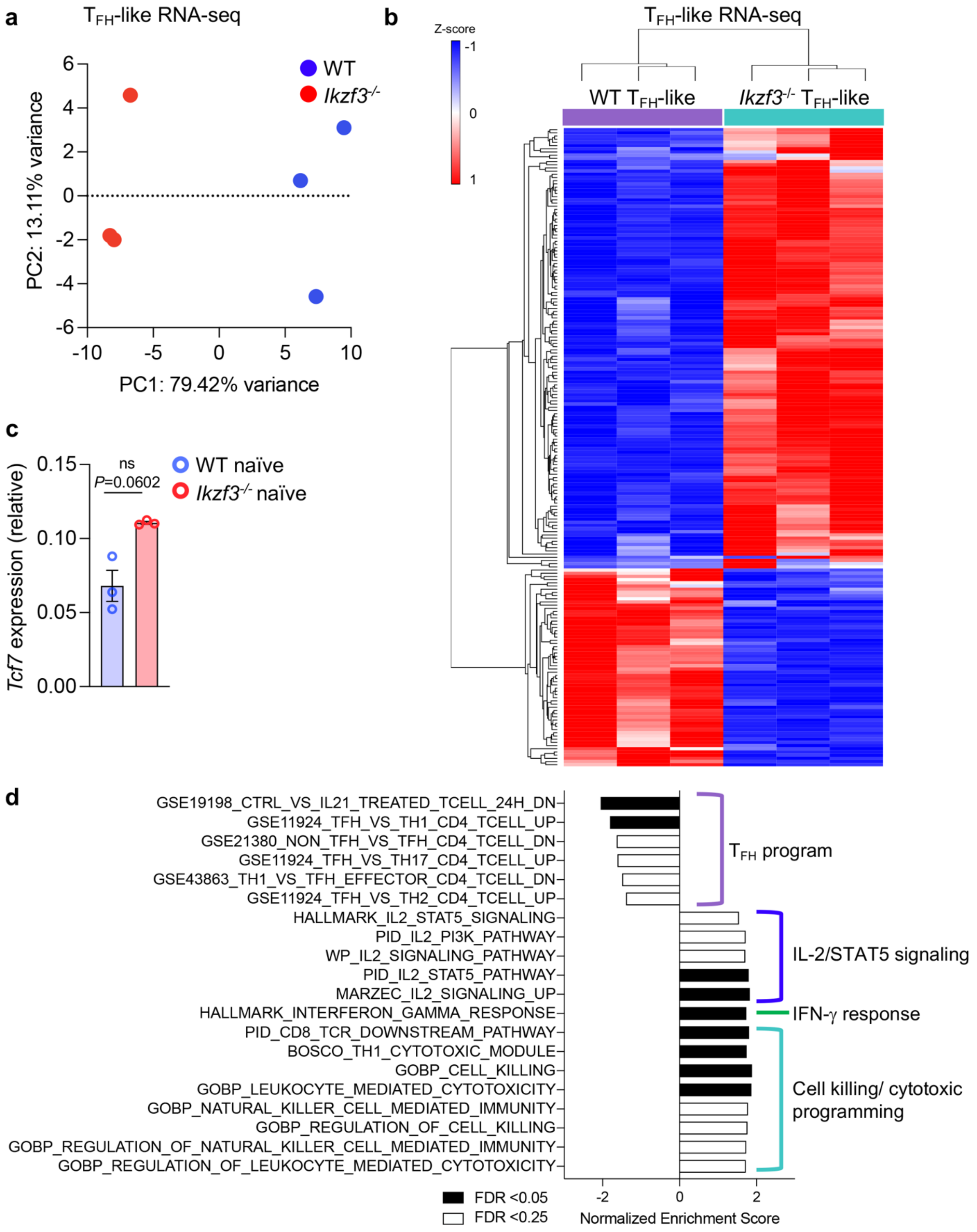

Supplementary Figure 4. Aiolos deficiency disrupts the T_{FH} gene program. (**a**,**b**,**d**) Naïve WT or Aiolos-deficient (*Ikzf3*$^{-/-}$) CD4^{+} T cells were cultured under T_{FH}-polarizing conditions for 3 days. RNA-seq analysis was performed to assess differentially expressed genes (DEGs) between WT and Aiolos-deficient cells. Data are compiled from 3 biological replicates from 3 independent experiments. (**a**) PCA analysis of DESeq2-normalized counts. (**b**) Heatmap displaying the top 200 significant DEGs. Clustering was performed using Euclidean distance. (**c**) Naïve WT or Aiolos-deficient (*Ikzf3*$^{-/-}$) CD4^{+} T cells were isolated via negative selection and *Tcf7* expression was assessed via qRT-PCR. Data are compiled from 3 independent experiments (n=3 ± s.e.m; two-sided, unpaired Student's t-test). (**d**) GSEA analysis of pre-ranked DEGs, compared against 'hallmark', 'curated', 'immunological signature', and 'gene ontology' gene sets. Source data are provided as a Source Data file.

Supplementary Figure 5

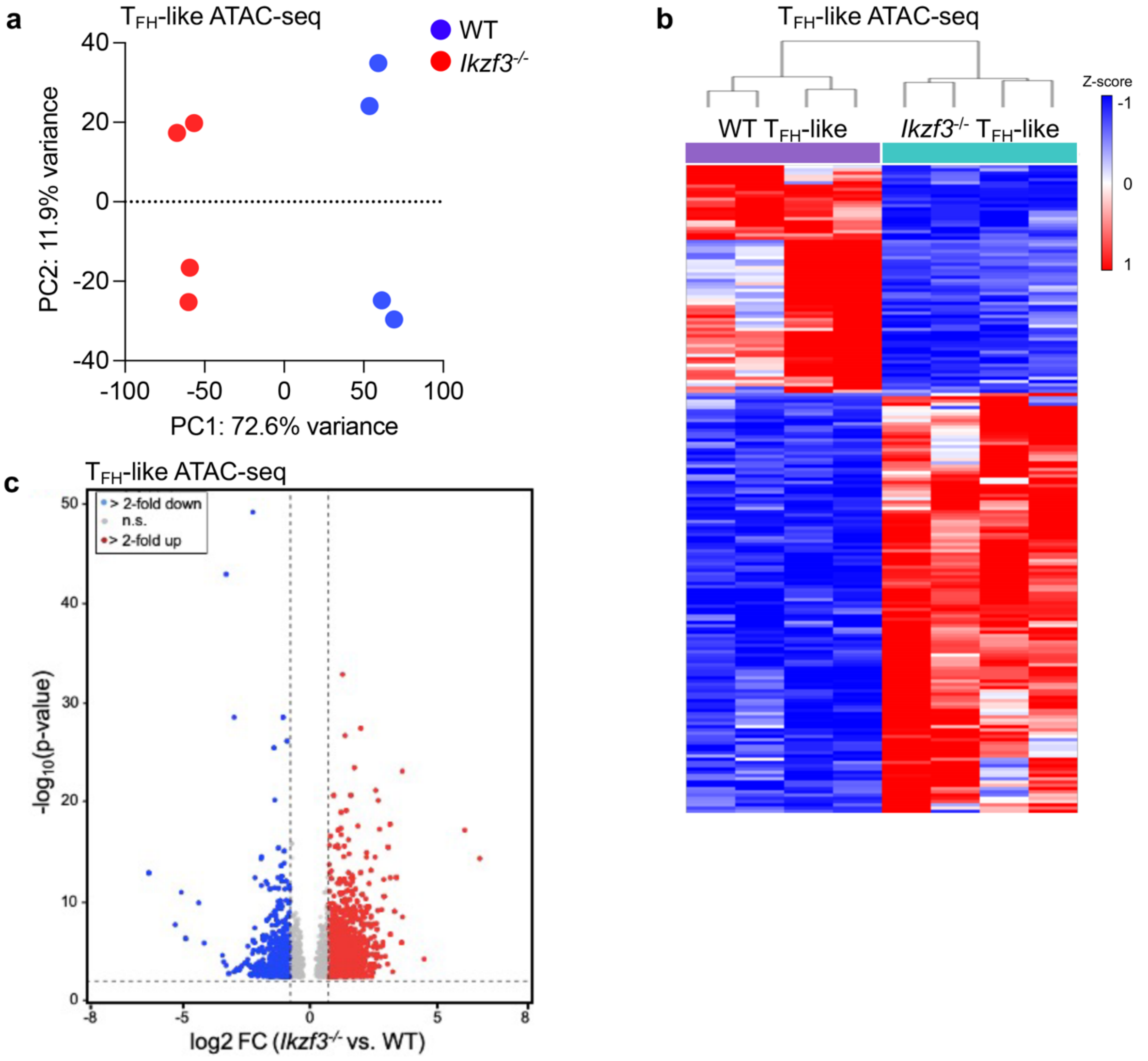

Supplementary Figure 5. Aiolos deficient T_{FH}-like cells exhibit global changes in chromatin accessibility. Naïve $CD4^+$ T cells from WT or *Ikzf3*$^{-/-}$ C57BL/6 mice were cultured under T_{FH}-polarizing conditions for 3 days. (**a**) PCA analysis was performed using normalized counts from DiffBind analysis. (**b**) Heatmap displaying the top 200 significant differentially accessible regions (DARs). Clustering was performed using Euclidean distance. Data are compiled from 4 biological replicates from 4 independent experiments. (**c**) Volcano plot displaying the top 10,000 significantly differentially accessible regions (DARs) in Aiolos-deficient vs WT cells. Regions were color-coded as follows: no significant changes in expression (gray), upregulated genes with >2-fold change in expression with a $P < 0.05$ (red), downregulated genes with >2-fold change in expression with a $P < 0.05$ (blue) ($n = 3$; two-sided DESeq2 analysis, as part of DiffBind package). Source data are provided as a Source Data file.

Supplementary Figure 6

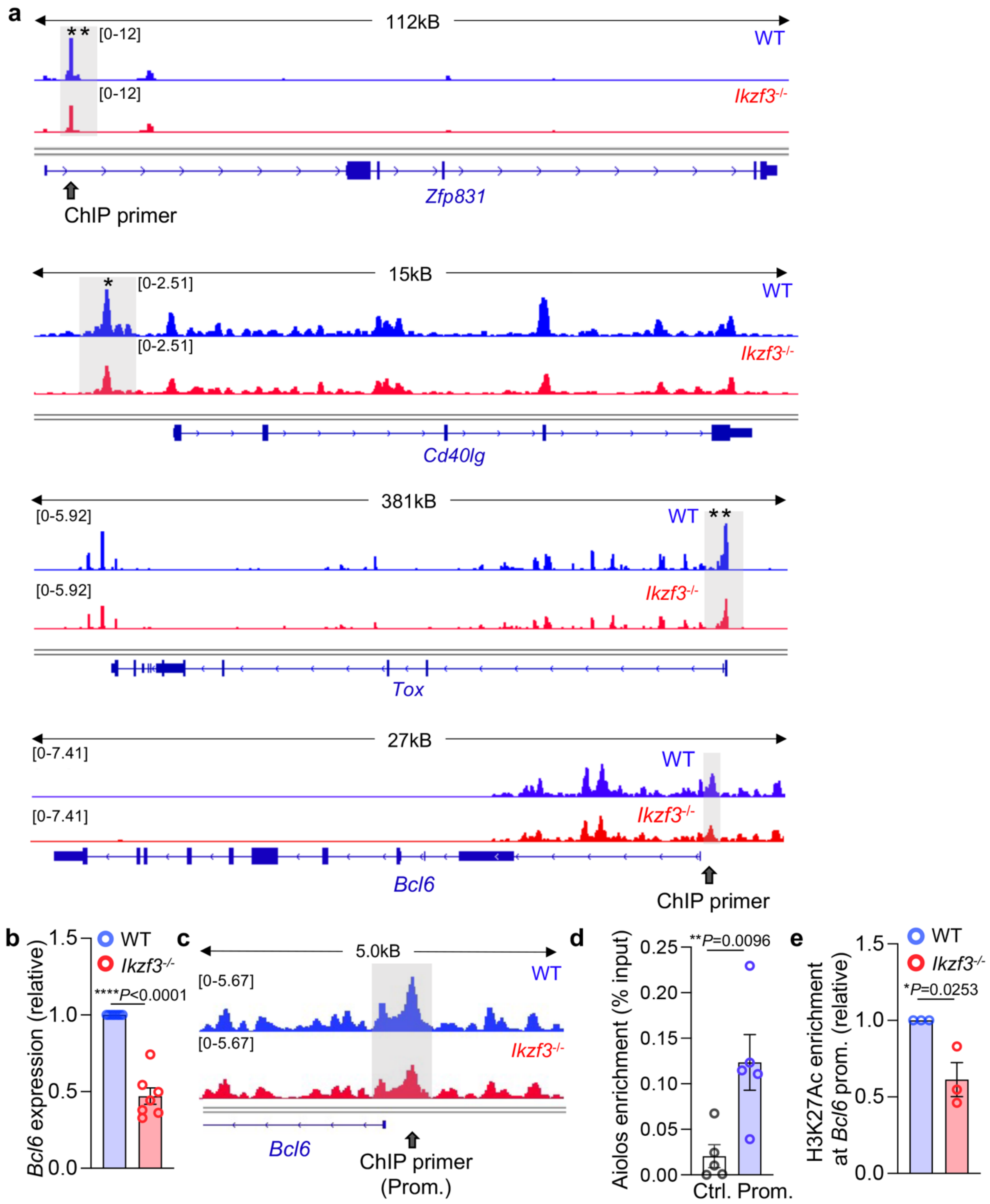

Supplementary Figure 6. Loss of Aiolos results in reduced accessibility at key T_{FH} gene regulatory regions. Naïve $CD4^+$ T cells from WT or *Ikzf3*$^{-/-}$ C57BL/6 mice were cultured under T_{FH}-polarizing conditions for 3 days. **(a)** Representative ATAC-seq analyses of the indicated loci are displayed as CPM-normalized Integrative Genomics Viewer (IGV) tracks. Asterisks indicate sites of statistically significant differences in accessibility between WT and Aiolos-deficient (*Ikzf3*$^{-/-}$) samples. Sites of interest are indicated with gray boxes. Where applicable, approximate ChIP primer locations are indicated with a gray arrow. Data are representative from 4 biological replicates from 4 independent experiments. (**b**) qRT-PCR analysis of *Bcl6* transcript. Data were normalized to *Rps18* control and are presented relative to the WT sample (n=7 ± s.e.m; ****P < 0.0001; two-sided, unpaired Student's t-test). (**c**) Representative ATAC-seq analyses of the partial *Bcl6* locus (from sample presented in 'A'). Sites of trending alterations accessibility, and approximate ChIP primer locations, are indicated. (**d**) ChIP analysis of Aiolos enrichment in WT T_{FH}-polarized cells at the indicated region, or negative control region, is shown. Data were normalized to total and presented as percent of input. Data are compiled from 4 independent experiments (n=5 ± s.e.m; **P < 0.01; two-sided, paired Student's t-test). (**e**) ChIP analysis of H3K27Ac enrichment at the *Bcl6* promoter (ChIP primer sites indicated) in WT versus *Ikzf3*$^{-/-}$ T_{FH}-polarized cells. Data were normalized to total input and presented as percent of input. Data are compiled from 3 independent experiments (n=3 ± s.e.m; *P < 0.05; two-sided, unpaired Student's t-test). Source data are provided as a Source Data file.

Supplementary Figure 7

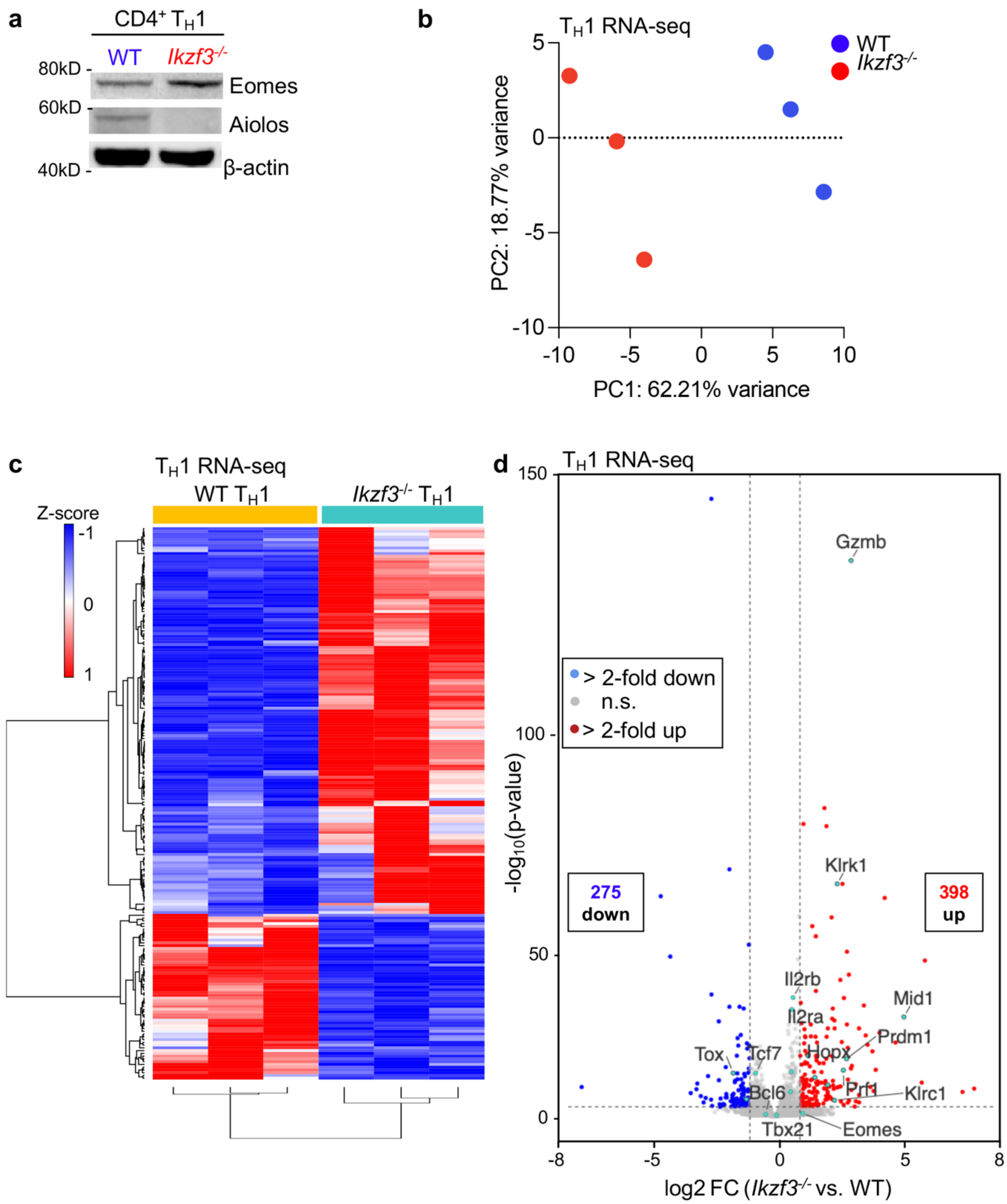

Supplementary Figure 7. Aiolos deficiency results in augmented expression of T_H1- and CTL-associated genes. Naïve WT or Aiolos-deficient (*Ikzf3$^{-/-}$*) $CD4^+$ T cells were cultured under T_H1-polarizing conditions for 3 days. (**a**) Immunoblot analysis of the indicated proteins. β-actin serves as a loading control. A representative image from 3 independent experiments is shown. (**b-d**) RNA-seq analysis was performed to assess differentially expressed genes (DEGs) between WT and Aiolos-deficient cells. (**b**) PCA analyses of normalized counts from T_H1 RNA-seq. (**c**) Heat-map of top 200 differentially expressed genes between WT and Aiolos KO samples. Changes in gene expression are presented as row (gene) Z-score from normalized counts. Genes were grouped by Euclidian Distance. (**d**) Volcano plot displaying gene expression changes between T_H1 polarized Aiolos-deficient vs WT cells; genes of particular interest are labeled. Genes were color-coded as follows: no significant changes in expression (gray), upregulated genes with >2-fold change in expression with a $P < 0.05$ (red), downregulated genes with >2-fold change in expression with a $P < 0.05$ (blue) ($n = 3$; two-sided DESeq2 analysis). Source data are provided as a Source Data file.

Supplementary Figure 8

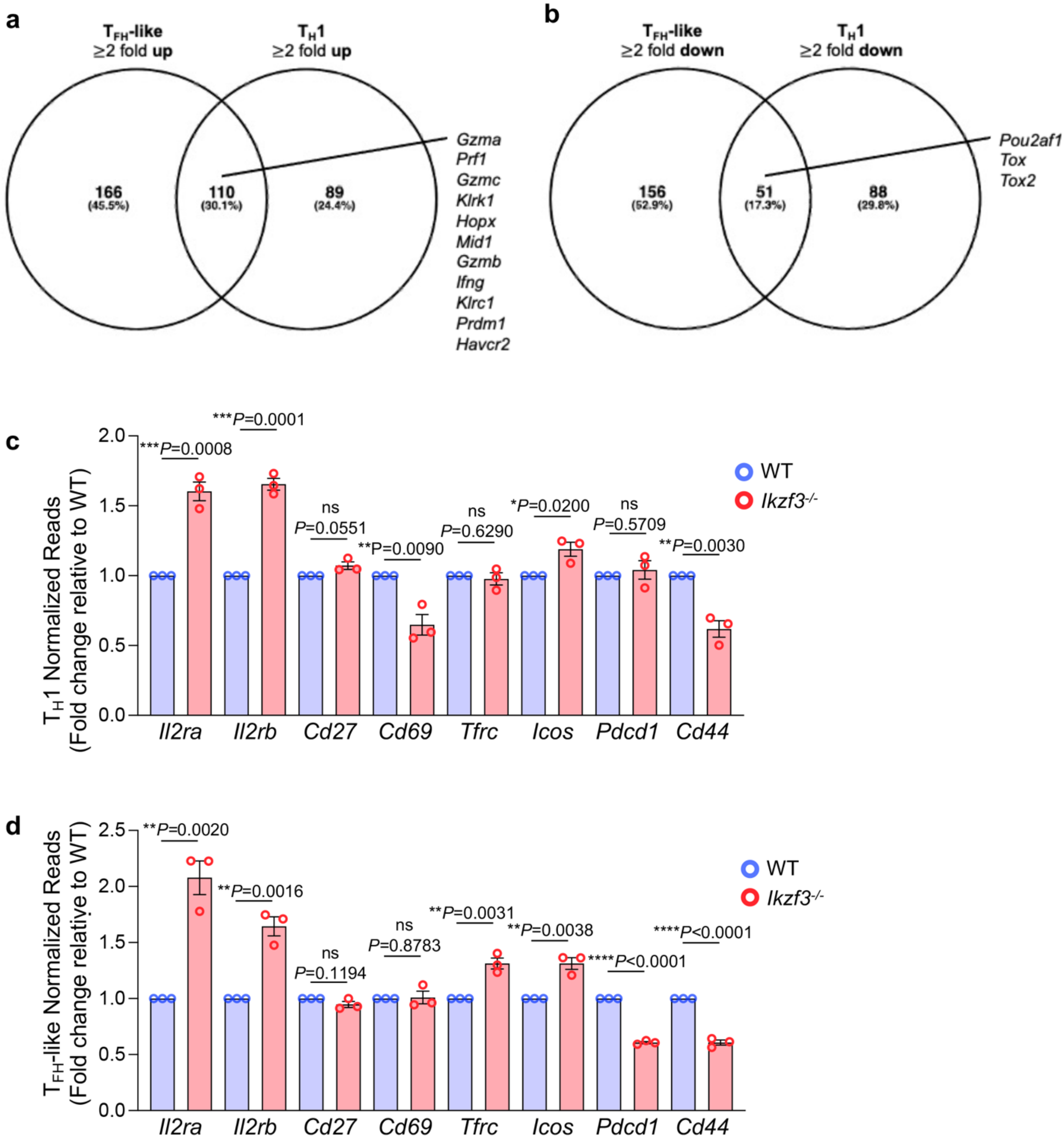

Supplementary Figure 8. Loss of Aiolos does not broadly impact activation-associated genes in T_H1 and T_{FH}-like cells. Naïve WT and Aiolos deficient cells were cultured under T_H1- or T_{FH}-polarizing conditions for 3 days and RNA-sequencing was performed. (**a-b**) Venn diagram of overlapping versus unique significant differentially expressed genes (DEGs) in WT vs Aiolos deficient T_{FH} and T_H1 populations. Unique and overlapping upregulated (**a**) and downregulated (**b**) DEGs are shown. (**c-d**) Normalized counts from RNA-seq of various activation markers in WT vs Aiolos deficient T_H1- (**c**) and T_{FH}- (**d**) polarized cells. Counts are presented relative to the WT sample for each gene. Data are compiled from 3 independent experiments per cell type (n=3 ± s.e.m; *P < 0.05, **P < 0.01, ***P < 0.001, ****P < 0.0001; two-sided, unpaired Student's t-test). Source data are provided as a Source Data file.

Supplementary Figure 9

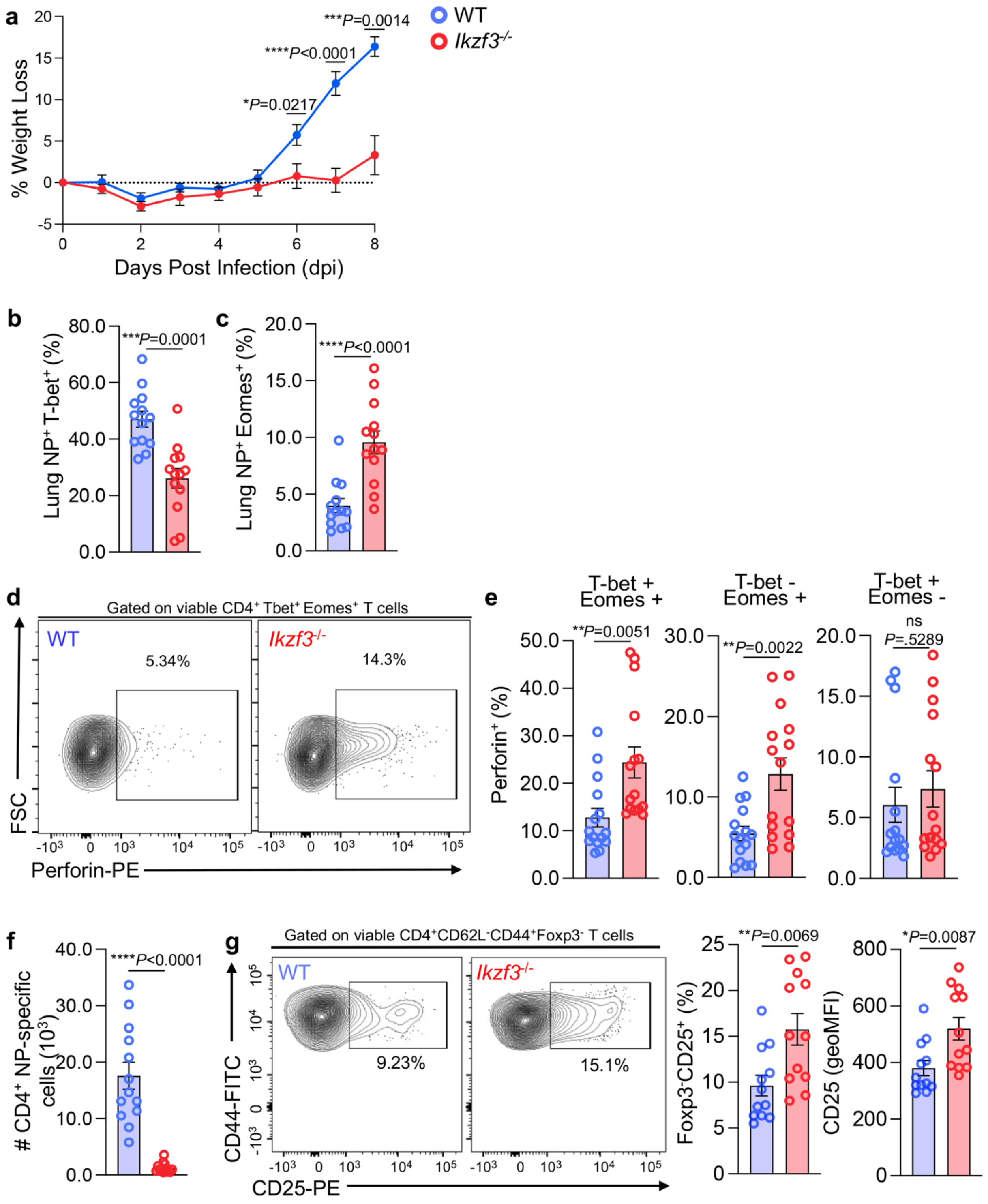

Supplementary Figure 9. Aiolos-deficient mice exhibit reduced weight loss, augmented CD4-CTL effector molecule production, and CD25 expresion during influenza virus infection. Naive WT and *Ikzf3*$^{-/-}$ mice were infected with PR8-influenza. On day 8 post-infection, DLN and lungs were harvested and samples were analyzed via flow cytometry. (**a**) Percent weightloss for WT versus *Ikzf3*$^{-/-}$ mice relative to intial weight on day of infection. Data are representative of 3 independent experiments n = 9 ± s.e.m; *P<0.05, ***P<0.001, ****P<0.0001; multiple two-sided, unpaired Student's t-tests). (**b-c**) Flow cytometry analyses of NP-specific CD4^{+} cells in the lung for T-bet (**b**) and Eomes (**c**) expression. Data are representative of 4 independent experiments (n=13 ± s.e.m; ****P* < 0.001, *****P*<0.0001; two-sided, unpaired Student's t-test). (**d-e**) DLN homogenates were cultured for 3 hours with protein transport inhibitors. Subsequently, expression of T-bet, Eomes, and perforin was assessed via flow cytometry. Data are compiled from 5 independent experiments (n=15 ± s.e.m**;** ***P*<0.01; two-sided, unpaired Student's t-test). (**f**) Enumeration of CD4^{+} NP-specific cells in the lung. Data are compiled from 4 independent experiments (n=13 ± s.e.m; *****P*<0.0001; two-sided, unpaired Student's t-test). (**g**) Flow cytometry analyses of CD25 surface expression on viable CD4^{+}CD44^{+}CD62L^{-}Foxp3^{-} (effector) T cells from the DLN. Percentage of CD25^{+} cells and median fluorescence intensity (MFI) are shown in bulk cell populations. Data are compiled from 4 independent experiments (n = 12 ± s.e.m; **P* < 0.05, ****P* <0.001; two-sided, unpaired Student's t-test). Source data are provided as a Source Data file.

Supplementary Figure 10

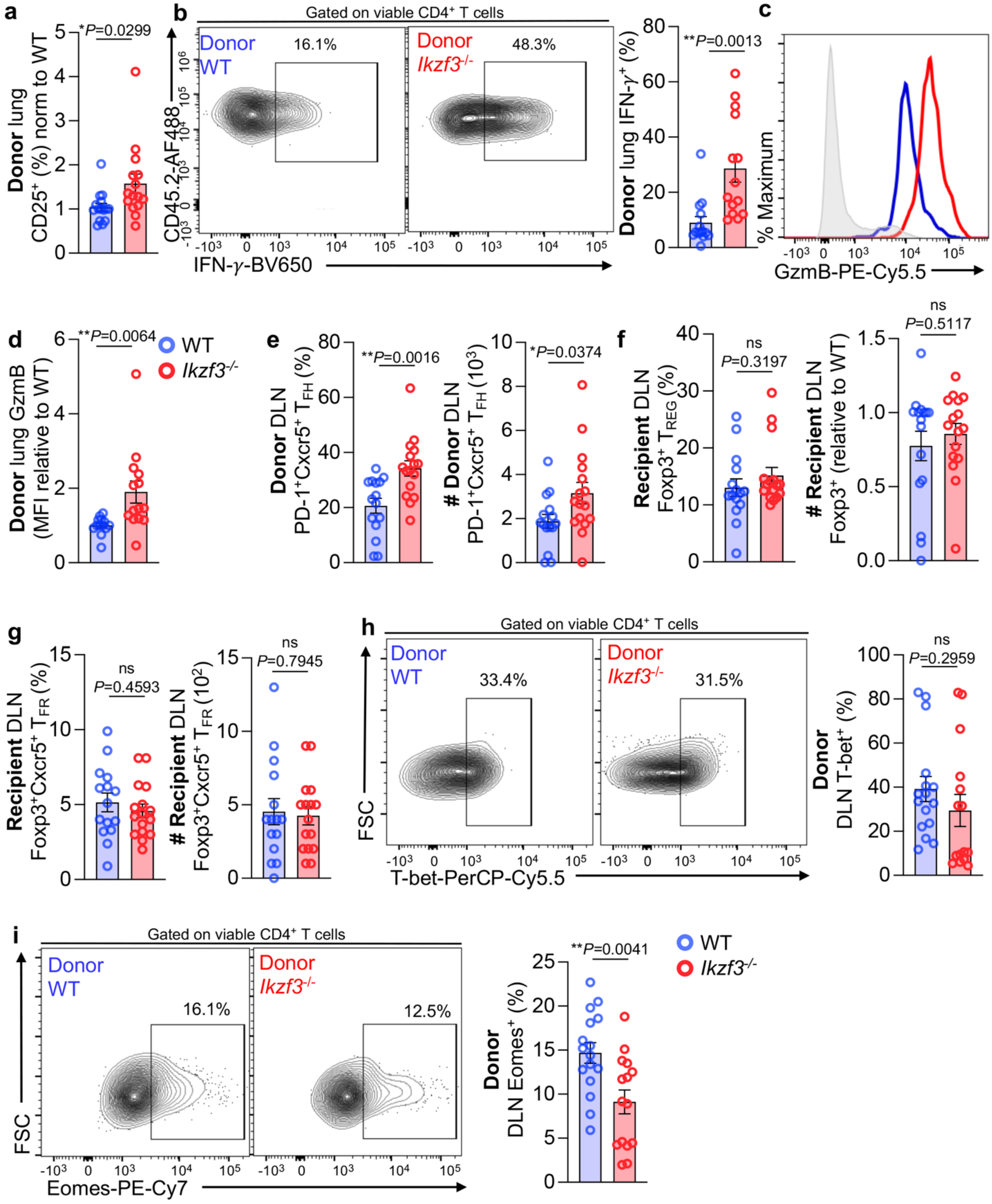

Supplementary Figure 10. Loss of Aiolos results in augmented CD4-CTL effector molecule expression in the lungs in a $CD4^+$ T cell-intrinsic manner. Naïve $CD4^+$ T cells from OT-II WT or OT-II-*Ikzf3*$^{-/-}$ mice were adoptively transferred into naïve CD45.1 recipients. After 24 hours, recipient mice were infected with 40 PFU $OVA_{323\text{-}339}$-expressing A/PR8/34 ("PR8-OT-II"). After 8 days, draining lymph nodes (DLN) and lungs were harvested and viable $CD45.2^+CD4^+$ populations were analyzed via flow cytometry. (**a**) Analysis of unstimulated donor cells from lung cell homogenates via flow cytometry for surface expression of CD25. Data are compiled from 4 independent experiments (n=15 ± s.e.m; **P*<0.05, two-sided, unpaired Student's t-test). (**b-d**) Cell homogenates from the lung were stimulated for 48 hours *ex vivo* with $OVA_{323\text{-}339}$ peptide. Suspensions were then incubated in the presence of protein transport inhibitors for 3 hours. Flow cytometry analyses was performed for IFN-g (**b**) and granzyme B (**c-d**) on donor cell populations. Data are compiled from 4 independent experiments (n = 14 ± s.e.m; ***P*<0.01, *****P*<0.0001, two-sided, unpaired Student's t-test). (**e**) Analysis of donor $PD\text{-}1^+Cxcr5^+$ T_{FH} populations in the DLN. Frequency and number of T_{FH} cells are shown. Data are compiled from 4 independent experiments (n=16 ± s.e.m; ***P*<0.01; two-sided, unpaired Student's t-test). (**f**) Analysis of recipient $Foxp3^+$ T_{REG} populations in the DLN. Frequency and number of T_{REG} cells are shown. To account for intra-experimental variability, counts were normalized to a single WT sample for each experiment. Data are compiled from 4 independent experiments (n=16 ± s.e.m; ***P*<0.01; two-sided, unpaired Student's t-test). (**g**) Analysis of recipient $Foxp3^+Cxcr5^+$ T_{FR} populations in the DLN. Frequency and number of T_{FR} cells are shown. Data are compiled from 4 independent experiments (n=15-16 ± s.e.m; ***P*<0.01; two-sided, unpaired Student's t-test). (**h-i**) Analysis of antigen-specific ($CD45.2^+CD4^+$) cells from DLN via flow cytometry for the expression of T-bet (**h**) and Eomes (**i**). Data are compiled from 5 independent experiments (n = 16 ± s.e.m; ***P*<0.01, two-sided, unpaired Student's t-test). Source data are provided as a Source Data file.

Supplementary Figure 11

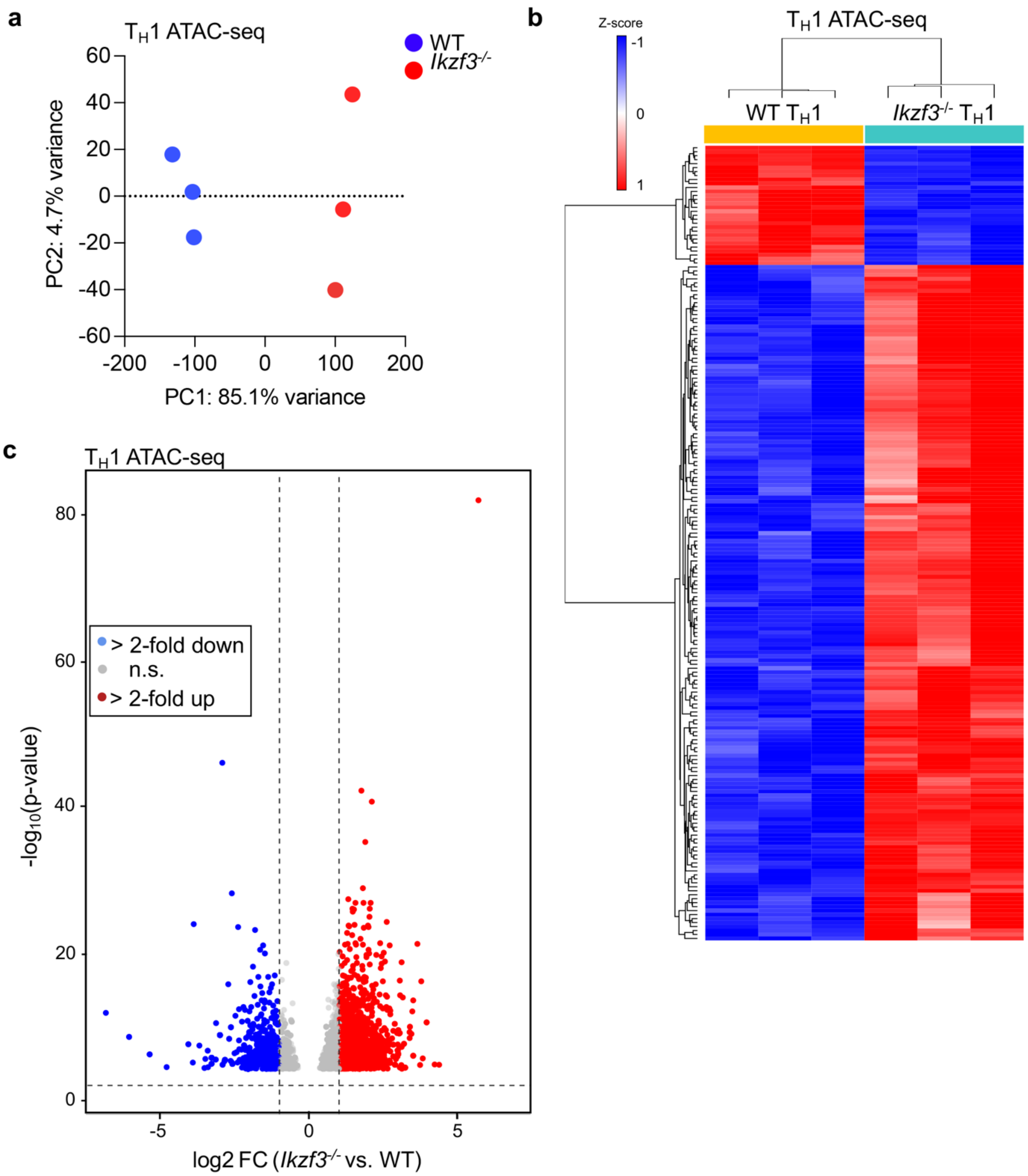

Supplementary Figure 11. Aiolos deficiency causes global alterations to the T_H1 transcriptome. WT and Aiolos-deficient cells were cultured under T_H1-polarizing conditions for 3 days and ATAC-seq was performed. Data shown are compiled from 3 independent experiments. (**a**) PCA analyses was performed using normalized counts from DiffBind. (**b**) Heatmap of top 200 significantly differently accessible regions. Changes in accessibility are presented as row (gene) Z-score and were grouped by Euclidian Distance. (**c**) Volcano plot displaying top 10,000 regions of altered accessibility between T_H1-polarized Aiolos-deficient vs. WT cells. Genes were color-coded as follows: no significant changes in accessibility (gray), regions with >2-fold increase in accessibility with a $P < 0.05$ (red), regions with >2-fold decrease in accessibility with a $P < 0.05$ (blue) ($n = 3$; two-sided DESeq2 analysis, as part of DiffBind package). Source data are provided as a Source Data file.

Supplementary Figure 12

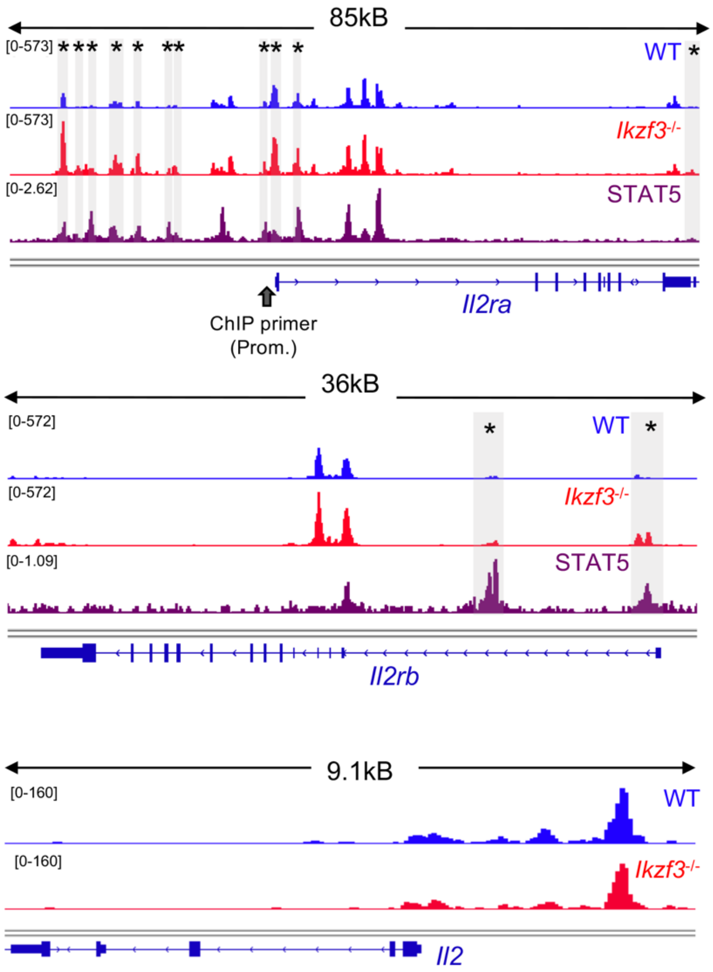

Supplementary Figure 12. Aiolos deficiency results in augmented accessibility at IL-2 receptor subunit gene loci, but not *Il2*. Naïve WT and Aiolos-deficient $CD4^+$ T cells were cultured for 3 days under T_H1-polarizing conditions. ATAC-seq analyses was performed and samples are displayed as CPM-normalized IGV tracks. Tracks are are overlaid with publicly available STAT5 ChIP-seq data (GSM1865310). Data are represented as follows: WT (blue, top track), Aiolos-deficient (red, middle track), and STAT5 ChIP-seq data alignment (dark purple, bottom track). Regulatory regions of significant differential accessibility are indicated by gray boxes with asterisks. Where applicable, approximate locations of primers used to assess STAT5 and H3K27Ac enrichment by ChIP are indicated with gray arrows. Data are representative from 3 independent experiments. Source data are provided as a Source Data file.

Supplementary Figure 13

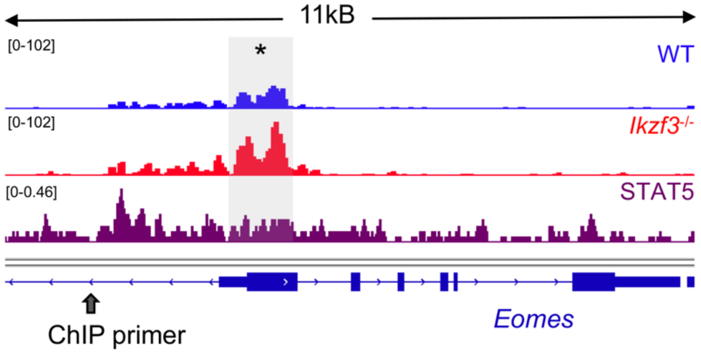

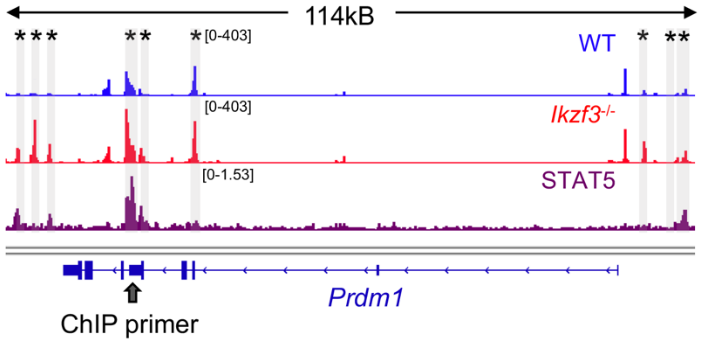

Supplementary Figure 13. Accessibility at key CD4-CTL transcription factor loci is augmented in the absence of Aiolos. Naïve WT and Aiolos-deficient $CD4^+$ T cells were cultured for 3 days under T_H1-polarizing conditions. ATAC-seq analyses was performed and samples are displayed as CPM-normalized IGV tracks. Tracks are are overlaid with publicly available STAT5 ChIP-seq data (GSM1865310). Data are represented as follows: WT (blue, top track), Aiolos-deficient (red, middle track), and STAT5 ChIP-seq data alignment (dark purple, bottom track). Regulatory regions of significant differential accessibility are indicated by gray boxes with asterisks. Where applicable, approximate locations of primers used to assess STAT5 and H3K27Ac enrichment by ChIP are indicated with gray arrows. Data are representative from 3 independent experiments. Source data are provided as a Source Data file.

Supplementary Figure 14

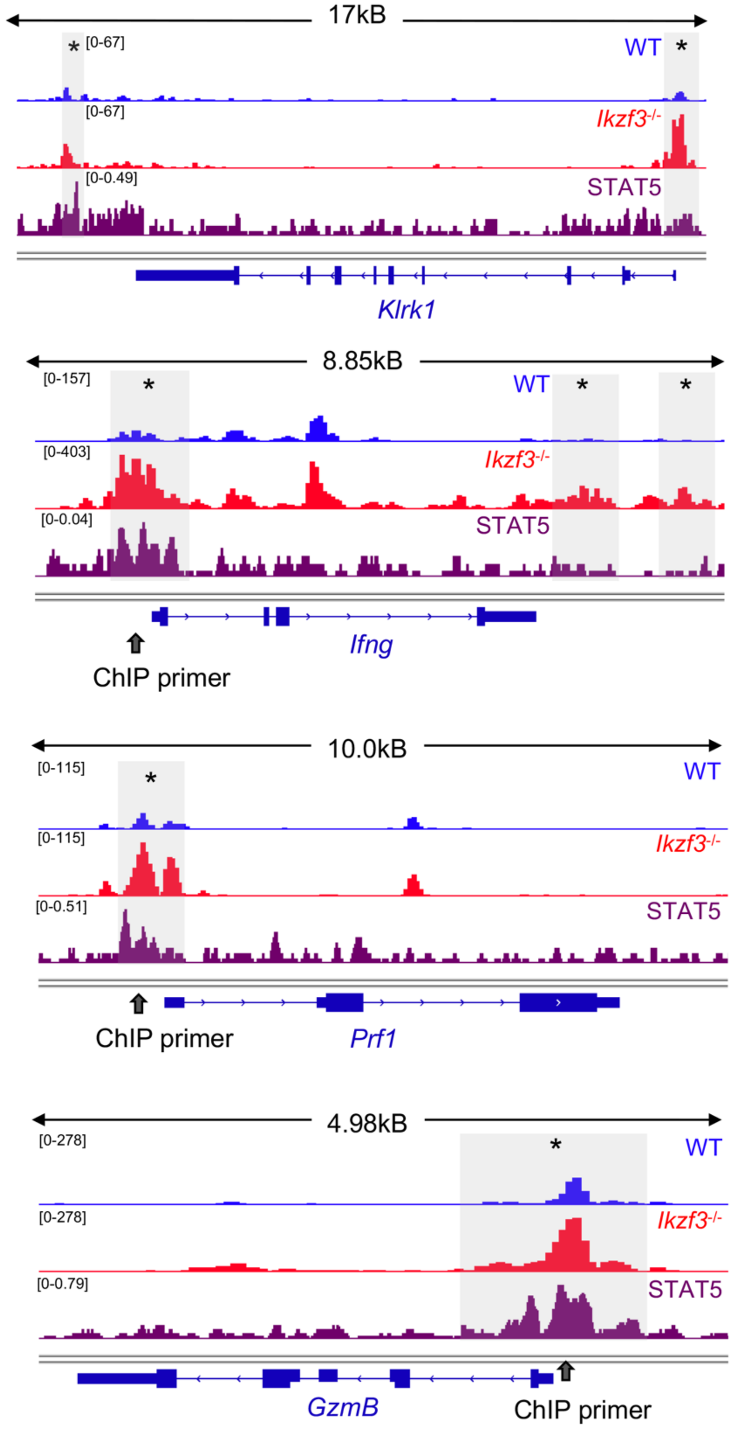

Supplementary Figure 14. Aiolos deficiency results in elevated accessibility at numerous CD4-CTL-associated gene loci. Naïve WT and Aiolos-deficient $CD4^+$ T cells were cultured for 3 days under T_H1-polarizing conditions. ATAC-seq analyses was performed and samples are displayed as CPM-normalized IGV tracks. Tracks are are overlaid with publicly available STAT5 ChIP-seq data (GSM1865310). Data are represented as follows: WT (blue, top track), Aiolos-deficient (red, middle track), and STAT5 ChIP-seq data alignment (dark purple, bottom track). Regulatory regions of significant differential accessibility are indicated by gray boxes with asterisks. Where applicable, approximate locations of primers used to assess STAT5 and H3K27Ac enrichment by ChIP are indicated with gray arrows. Data are representative from 3 independent experiments. Source data are provided as a Source Data file.

Supplementary Figure 15

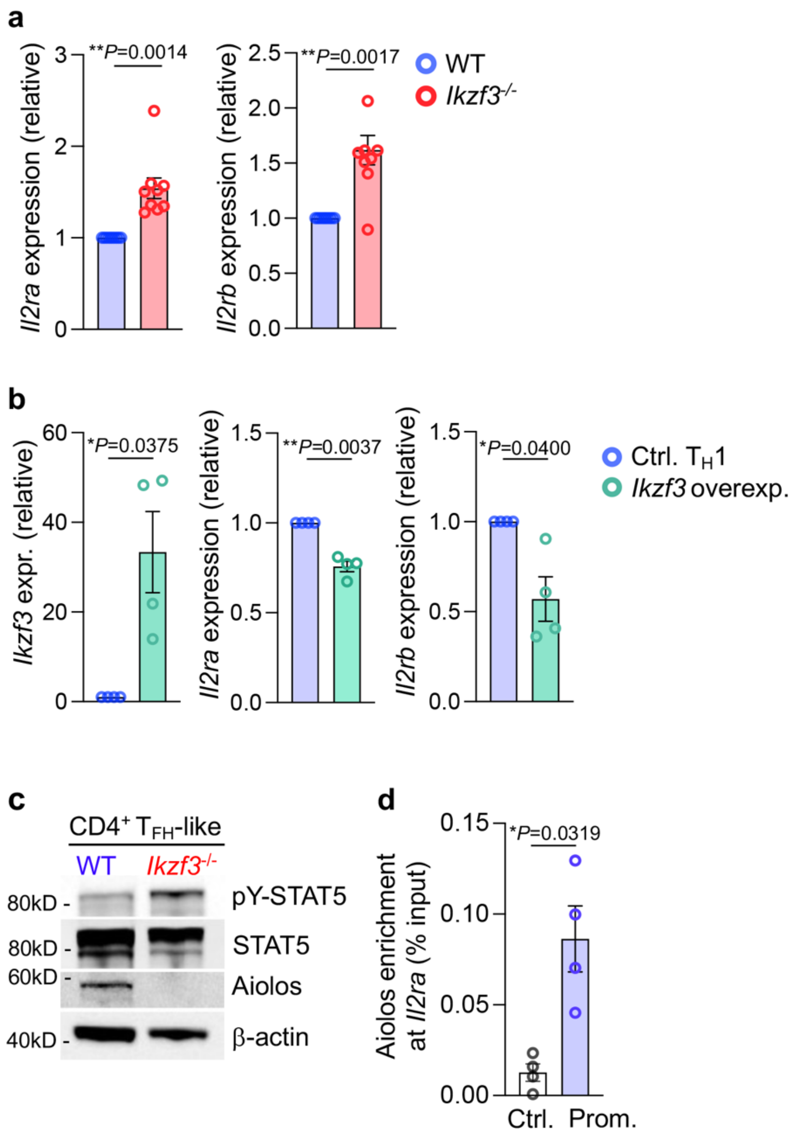

Supplementary Figure 15. Aiolos negatively regulates IL-2 responsiveness. (**a**) Naïve WT or Aiolos-deficient $CD4^+$ T cells were cultured under T_H1 polarizing conditions for 3 days. Transcript analyses of *Il2ra* and *Il2rb* was performed via qRT-PCR. Data were normalized to *Rps18* control and are presented relative to WT. Data are compiled from 6 independent experiments (n=9 ± s.e.m; $^{**}P < 0.01$, two-sided, unpaired Student's t-test). (**b**) Naïve WT $CD4^+$ T cells were retrovirally transduced with the gene encoding Aiolos or empty vector control and cultured under T_H1-polarizing conditions for 3 days. Transcript analysis of the indicated genes was performed via qRT-PCR. Data are normalized to *Rps18* control and presented as relative to the empty vector control (Ctrl. T_H1). Data are compiled from 4 independent experiments (n=4 ± s.e.m; *P<0.05, **P<0.01; two-sided, paired Student's t-test). (**c-d**) Naïve $CD4^+$ T cells from WT or $Ikzf3^{-/-}$ C57BL/6 mice were cultured under T_{FH}-polarizing conditions for 3 days. (**c**) Analysis of indicated proteins via immunoblot with b-actin as a loading control. A representative image from 2 independent experiments is shown. (**d**) ChIP analysis of Aiolos enrichment at the *Il2ra* promoter, or negative control region, is shown. Data were normalized to total input and are presented as percent of input minus IgG background. Data are compiled from 4 independent experiments (n=5 ± s.e.m; $^{*}P < 0.05$; two-sided, paired Student's t-test). Source data are provided as a Source Data file.

Supplementary Figure 16

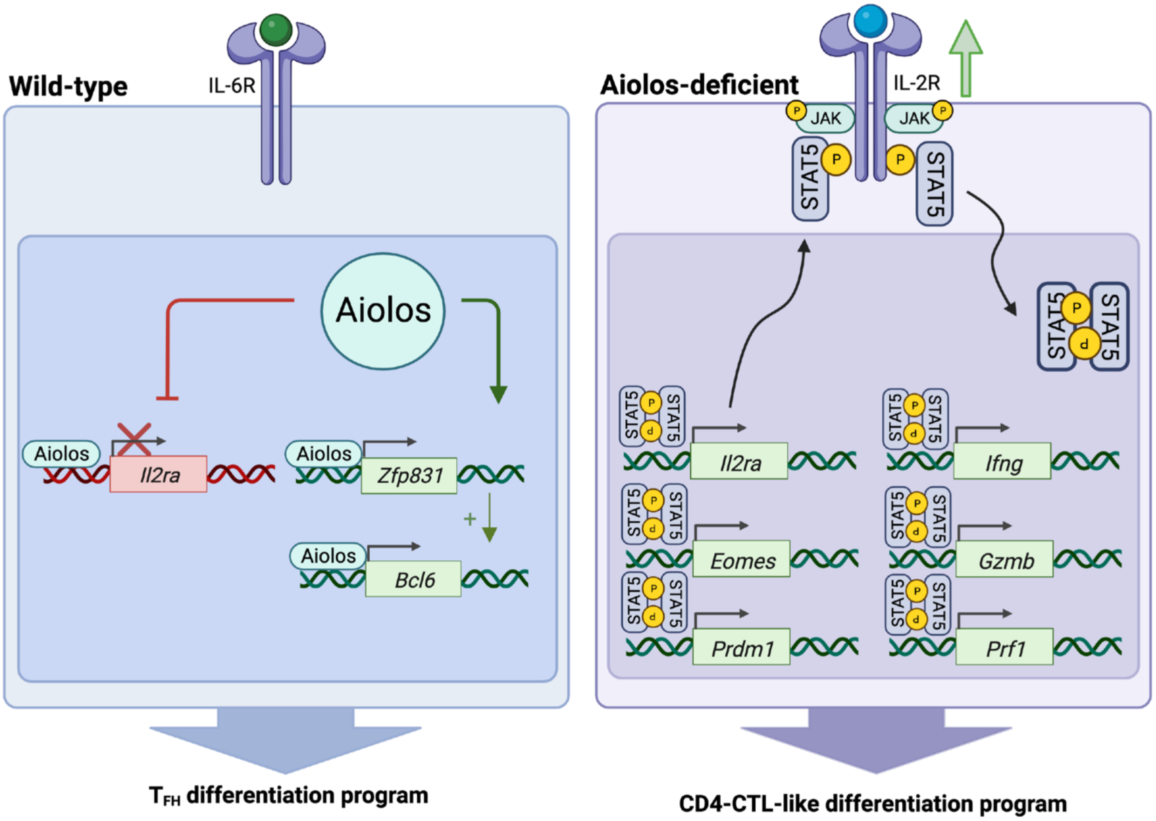

Supplementary Figure 16. Schematic depicting the mechanisms by which Aiolos reciprocally regulates the T_{FH} and CD4-CTL-like transcriptional programs. Figure created with BioRender.com.

Supplementary Uncropped Immunoblots:

Supplementary Fig. 3b

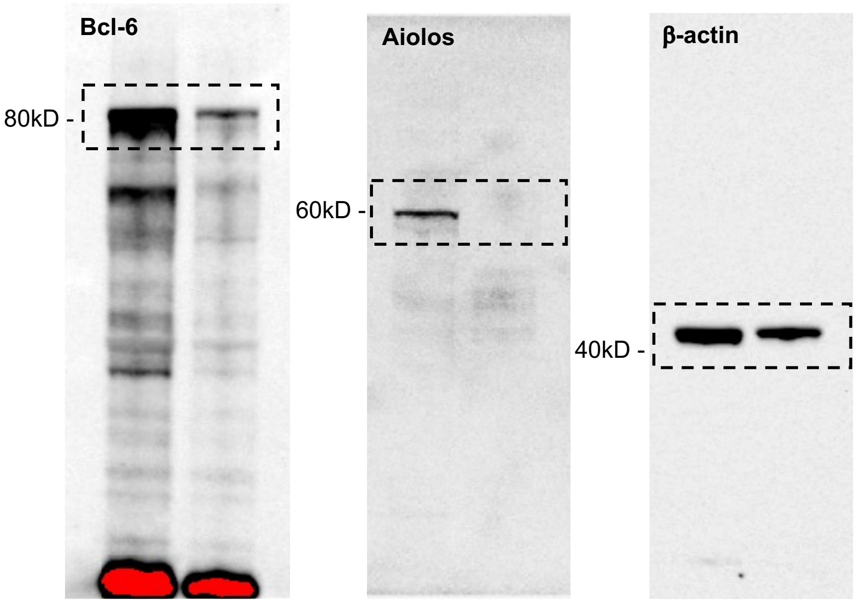

Supplementary Fig. 7a

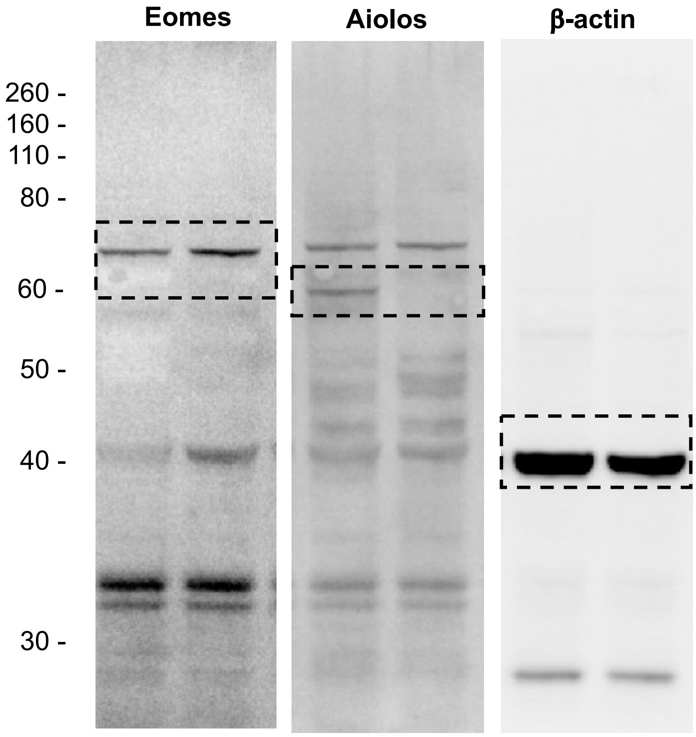

Supplementary Fig. 15c

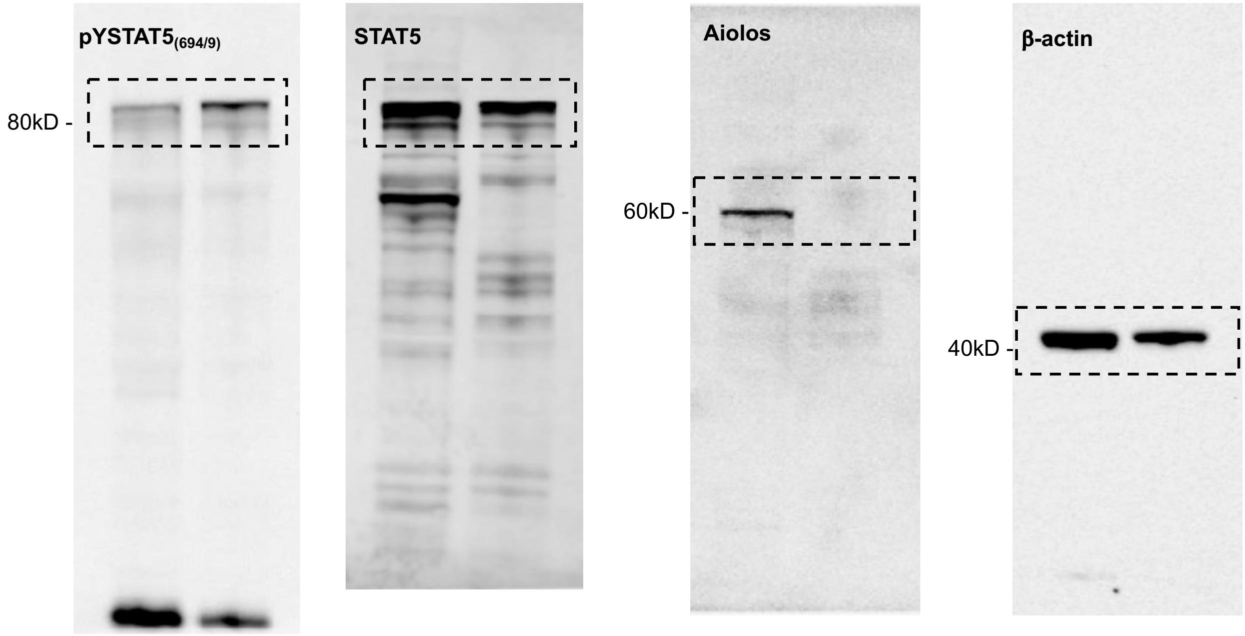

Supplement: Supplementary file 1 — Supplementary Information [file 41467_2023_37420_MOESM1_ESM.pdf]
