# Supplementary material for: Aiolos represses CD4+ T cell cytotoxic programming via reciprocal regulation of TFH transcription factors and IL-2 sensitivity
Source: Nat Commun. 2023 Mar 24;14:1652. doi: 10.1038/s41467-023-37420-0 (PMC10039023; doi:10.1038/s41467-023-37420-0)
Supplement: Supplementary file 3 — Reporting Summary [file 41467_2023_37420_MOESM3_ESM.pdf]

nature portfolio

Corresponding author(s): Kenneth J. Oestreich

Last updated by author(s): 2-22-2023

Reporting Summary

Nature Portfolio wishes to improve the reproducibility of the work that we publish. This form provides structure for consistency and transparency in reporting. For further information on Nature Portfolio policies, see our Editorial Policies and the Editorial Policy Checklist.

Statistics

For all statistical analyses, confirm that the following items are present in the figure legend, table legend, main text, or Methods section.

n/a	Confirmed	
☐	☒	The exact sample size (n) for each experimental group/condition, given as a discrete number and unit of measurement
☐	☒	A statement on whether measurements were taken from distinct samples or whether the same sample was measured repeatedly
☐	☒	The statistical test(s) used AND whether they are one- or two-sided<br>*Only common tests should be described solely by name; describe more complex techniques in the Methods section.*
☐	☒	A description of all covariates tested
☐	☒	A description of any assumptions or corrections, such as tests of normality and adjustment for multiple comparisons
☐	☒	A full description of the statistical parameters including central tendency (e.g. means) or other basic estimates (e.g. regression coefficient) AND variation (e.g. standard deviation) or associated estimates of uncertainty (e.g. confidence intervals)
☐	☒	For null hypothesis testing, the test statistic (e.g. F, t, r) with confidence intervals, effect sizes, degrees of freedom and P value noted<br>*Give P values as exact values whenever suitable.*
☒	☐	For Bayesian analysis, information on the choice of priors and Markov chain Monte Carlo settings
☒	☐	For hierarchical and complex designs, identification of the appropriate level for tests and full reporting of outcomes
☒	☐	Estimates of effect sizes (e.g. Cohen's d, Pearson's r), indicating how they were calculated

Our web collection on statistics for biologists contains articles on many of the points above.

Software and code

Policy information about availability of computer code

Data collection	Data were collected utilizing the following open-source or commercially available software programs: BD FACSDiva (8.0.2), BioRad Image Lab (6.0.1 build 34), BioRad CFX Manager (3.1). ATAC-seq data was generated by Illumina Novaseq SP. RNA-seq was performed by Azenta Life Sciences (formerly Genewiz).
Data analysis	Analyses were conducted using the Microsoft Office Suite, version 16.43 (including Microsoft Word, Microsoft Excel, and Microsoft Powerpoint), BD FlowJo (version 10.8.1), and open-source software, including tools available on Galaxy (http://usegalaxy.org): Bowtie2 (2.4.4), MACS2, Integrative Genomics Viewer (version 2.9.4), Morpheus (Broad Institute), VolcanoseR (https://huygens.science.uva.nl/VolcaNoseR/; PMID: 33239692), and ClustVis (https://bio.tools/clustvis). Data preparation for this manuscript did not require the use of custom code or software. The final schematic (Extended Data Fig. 14) was generated using BioRender (https://biorender.com/).<br><br>All statistical analyses were performed using the GraphPad Prism software (version 9.3.1). For single comparisons, two-tailed Student's t-tests (paired or unpaired, as noted) were performed. For multiple comparisons, one-way ANOVA with Tukey's multiple comparison tests were performed. Error bars indicate the standard error of the mean. P values <0.05 were considered statistically significant.<br><br>ATAC-seq data was generated by Illumina Novaseq SP. Resultant sequences were trimmed and aligned to mm10 using Bowtie2. After trimming, all subsequent analyses were performed utilizing the indicated tools in Galaxy (usegalaxy.org). Samples were filtered by read quality (>30), as well as to remove duplicates and mitochondrial reads. Statistically significant peaks were identified using MACS2 callpeak. DiffBind was used to identify regions of significant differential accessibility between WT and Aiolos-deficient samples for each cell type. Regions with adjusted P values <0.05 were considered statistically significant. CPM-normalized tracks were visualized using Integrative Genomics Viewer (IGV) version 2.12.2. PCA plots were generalized using normalized counts from DiffBind and Clustvis software (https://bio.tools/clustvis). Motif

analyses were performed using regions of significantly altered accessibility Aiolos-deficient samples relative to WT using HOMER (version version 4.11; http://homer.ucsd.edu/homer/motif/). Heatmap generation and clustering (by Euclidean distance) were performed using normalized counts from DiffBind analyses and the Morpheus software (https://software.broadinstitute.org/morpheus). Volcano plots were generated using -log10(adjusted p-value) and log2 fold change values from DiffBind analyses and VolcaNoseR software (https://huygens.science.uva.nl/VolcaNoseR/)

RNA-seq was performed by Azenta Life Sciences (formerly Genewiz). Samples were provided to Azenta Life Sciences for polyA selection, library preparation, sequencing, and DESeq2 analysis (3 biological replicates per cell type and genotype, from 3 independent experiments). Genes with a p-value < 0.05 were considered significant, and those with fold changes of >1.5 were defined as differentially expressed genes (DEGs) for each comparison in the present study. Genes pre-ranked by multiplying the sign of the fold-change by -log10 (p-value) were analyzed using the Broad Institute Gene Set Enrichment Analysis (GSEA) software for comparison against 'hallmark', 'gene ontology', and 'immunological signature' gene sets. Heatmap generation and clustering (by Euclidean distance) were performed using normalized log2 counts from DEseq2 analysis and the Morpheus software (https://software.broadinstitute.org/morpheus). Volcano plots were generated using -log10(p-value) and log2 fold change values from DEseq2 analysis and VolcaNoseR software (https://huygens.science.uva.nl/VolcaNoseR/)

For manuscripts utilizing custom algorithms or software that are central to the research but not yet described in published literature, software must be made available to editors and reviewers. We strongly encourage code deposition in a community repository (e.g. GitHub). See the Nature Portfolio guidelines for submitting code & software for further information.

Data

Policy information about availability of data

All manuscripts must include a data availability statement. This statement should provide the following information, where applicable:

- Accession codes, unique identifiers, or web links for publicly available datasets
- A description of any restrictions on data availability
- For clinical datasets or third party data, please ensure that the statement adheres to our policy

Data and material availability: RNA- and ATAC-seq data sets have been deposited in the GEO repository under accession number GSE203066. Publicly available data under GSE58597 and GSM1865310 were also analyzed for use in this study. Source data are provided with this paper. All other materials from this study will be made available upon reasonable request. Requests should be sent to the corresponding author.

Human research participants

Policy information about studies involving human research participants and Sex and Gender in Research.

Reporting on sex and gender	For human tissue samples, de-identified pediatric tonsillar tissues were obtained from the Comprehensive Human Tissue Network- Western Division according to IRB Protocol #2003H0194, and thus are not considered human research participants. As samples were completely de-identified, reporting on/analyzing sex as a co-variate was not possible.
Population characteristics	As samples were completely de-identified, reporting on/analyzing population characteristics was not available.
Recruitment	De-identified pediatric tonsillar tissues were obtained from the Comprehensive Human Tissue Network- Western Division
Ethics oversight	Materials were collected and utilized in accordance with protocols approved by the Ohio State University Institutional Review Board (IRB Protocol #2003H0194). Donor consent was acquired for all samples.

Note that full information on the approval of the study protocol must also be provided in the manuscript.

Field-specific reporting

Please select the one below that is the best fit for your research. If you are not sure, read the appropriate sections before making your selection.

[x] Life sciences [ ] Behavioural & social sciences [ ] Ecological, evolutionary & environmental sciences

For a reference copy of the document with all sections, see nature.com/documents/nr-reporting-summary-flat.pdf

Life sciences study design

All studies must disclose on these points even when the disclosure is negative.

Sample size	For all analyses, a minimum of n=3 was chosen because it was the minimal replicate number sufficient to ascertain statistics by t-test or one-way ANOVA.
Data exclusions	One data point from Fig. 1D exhibited a significantly elevated number of overall antigen-specific CD4+ T cells relative to other WT and Aiolos KO samples (noted in source data). This individual data point was determined to be a mathematical outlier using the ROUT test (Q=1%) in GraphPad Prism. This sample was therefore excluded from Tfh cell analyses. However, it should be noted that the observed differences were

	statistically significant whether this data point was included or not.
Replication	All experiments (ATAC-seq, ChIP, qRT-PCR, RNA-sequencing, flow cytometry analyses, etc.) were performed at least three times with distinct biological replicates and replicated successfully. Immunoblot analyses were repeated at least two times with completely independent biological replicates. Replicates have been noted in all figure legends.
Randomization	As our study targeted specific mice (or cells derived from these mice) of known genotypes, randomization was not possible. Covariates, such as animal sex (matched within each experiment/replicate), age (matched within each experiment/replicate), and breeding facility; and cell culture conditions were kept constant. For human tissue samples, de-identified pediatric tonsillar tissues were obtained from the Comprehensive Human Tissue Network- Western Division. All samples were processed and stained in the same manner for the analysis of Aiolos protein expression in naive, non-Tfh, and Tfh populations, and thus randomization was not required.
Blinding	Blinding was not possible in this study due to the nature of the treatments and the fact that experimenters carried out both treatments and analyses. However, analyses were performed and/or verified by at least three independent researchers.

Reporting for specific materials, systems and methods

We require information from authors about some types of materials, experimental systems and methods used in many studies. Here, indicate whether each material, system or method listed is relevant to your study. If you are not sure if a list item applies to your research, read the appropriate section before selecting a response.

Materials & experimental systems

n/a	Involved in the study
☐	☒ Antibodies
☐	☒ Eukaryotic cell lines
☒	☐ Palaeontology and archaeology
☐	☒ Animals and other organisms
☒	☐ Clinical data
☒	☐ Dual use research of concern

Methods

n/a	Involved in the study
☒	☐ ChIP-seq
☐	☒ Flow cytometry
☒	☐ MRI-based neuroimaging

Antibodies

Antibodies used

Mouse sample flow cytometry:
Example catalog numbers are provided for each antibody; catalog numbers will vary based on fluorochrome and volume of product:
anti-CD4 (clone GK1.5; R&D Systems) - Catalog # for anti-CD4:AF488: FAB554G
anti-CD44 (clone IM7; BD Biosciences) - Catalog # for anti-CD44:V450: 560451
anti-CD62L (clone MEL-14; ThermoFisher) - Catalog # for anti-CD62L-APC-eFluor780: 47-0621-82
anti-PD-1 (clone 29F.1A12; BioLegend) - Catalog # for anti-PE-Cy7: 135216
anti-Cxcr5 (clone SPRCL5; ThermoFisher) - Catalog # for anti-Cxcr5:PE:12-7185-82
anti-CD25 (clone PC61.5; ThermoFisher) - Catalog # for anti-CD25:PE: 12-0251-82
anti-T-bet (clone 4B10; BioLegend) - Catalog # for anti-T-bet:PerCP-Cy5.5: 644806
anti-Foxp3 (clone FJK-16s; ThermoFisher) - Catalog # for anti-Foxp3:PerCP-Cy5.5: 45-5773-82
anti-Bcl-6 (clone K112-9; BD Biosciences) - Catalog # for anti-Bcl-6:AF488: 561524
anti-IL-2 (clone JES6-5H4; BioLegend) - Catalog # for anti-IL-2:APC: 503810
anti-Eomes (clone DAN11MAG; ThermoFisher) - Catalog # for anti-Eomes:PE-Cy7: 25-4875-82
anti-Granzyme B (clone GB11; ThermoFisher) - Catalog # for anti-Granzyme B:PE: GRB04
anti-Perforin (clone S16009A; BioLegend) - Catalog # for anti-Perforin:APC: 154304
anti-CD45.2 (1:100; clone 104; BioLegend) - Catalog # for anti-CD45.2:AF488: 109816
anti-CD122 (1:50; clone 5H4; Biolegend) - Catalog # for anti-CD122:BUV711: B741537
anti-NKG2A/C/E (1:100; clone 20D5; Biolegend) - Catalog # for anti-NKG2A/C/E:BUV737: B741808
anti-Aiolos (1:50; clone S48-791; BD Biosciences) - Catalog # for anti-Aiolos:AF488: 565266
anti-IFNg (1:400; XM1.2, Biolegend) - Catalog # for anti-IFNg:BV650: 505831
anti-CD45R/B220 (1:300; clone RA3-6B2; Biolegend) - Catalog # for anti-D45R/B220:BV510: 103247
CD11b (1:300; clone M1/70; Biolegend) - Catalog # for anti-CD11b:BV510: 101245
F4/80 (1:300, clone BM8, Biolegend) - Catalog # for anti-F4/80:BV510: 123135
CD11c (1:300, clone N418, Biolegend) - Catalog # for anti-CD11c:BV510: 117337

Human sample flow cytometry:
anti-CD44:APC (clone DB105, Miltenyi Biotec) - Catalog # 130-110-294
anti-CD4:AF700 (clone RPA-T4, Thermo Fisher Scientific) - Catalog # 56-00049-42
anti-CD45RA:APC-Cy7 (clone REA1047, Miletnyi Biotec) - Catalog # 130-117-747
anti-AIOLOS:PE (clone 16D9C97, BioLegend) - Catalog # 371104
anti-CXCR5:PE-Vio770 (clone MU5UBEE, Thermo Fisher Scientific) - Catalog # 25-9185-42
anti-FOXP3:PerCP-Vio770 (clone PCH101, Thermo Fisher Scientific) - Catalog # 45-4776-42
anti-CD3:PE-Vio615 (clone UCHT1, BioLegend) - Catalog # 300450

anti-CD8:FITC (clone REA734, Miltenyi Biotec) - Catalog # 130-110-677
anti-PD-1:BV421 (clone MIH4, BD Biosciences) - Catalog # 564323
anti-CD62L:BV605 (clone DREG-56, BD Biosciences) - Catalog # 562720
anti-CD45RO:BV650 (clone UCHTL1, Biolegend) - Catalog # 304232

Chromatin immunoprecipitation analyses:
anti-Aiolos (clone D1C1E, Cell Signaling) - Catalog # 15103
anti-STAT5 (polyclonal, R&D Systems) - Catalog # AF2168
Rabbit Anti-Mouse IgG H&L (isotype control, Abcam) - Catalog # ab6709

Immunoblot analyses:
anti-Aiolos (clone D1C1E, Cell Signaling) - Catalog # 15103
anti-Bcl-6 (clone K112, BD Biosciences) - Catalog # 561520
anti-pSTAT5(Y694/9) (clone 47, BD Biosciences) - Catalog # 611964
anti-STAT5 (clone D206Y, Cell Signaling) - Catalog # 94205
anti-beta-Actin:HRP (clone 2D1D10, GenScript) - Catalog # A00730
anti-Eomes (polyclonal, Abcam 1:5,000) - Catalog # ab23345
goat anti-mouse:HRP (Jackson Immunoresearch) - Catalog # 115-035-003
mouse anti-rabbit:HRP (Santa Cruz Biotechnology) - Catalog # sc-2357

serum ELISA analysis antibodies:
Biotinylated goat-anti-mouse IgM (polyclonal, Southern Biotech) - Catalog # 1021-08.
Biotinylated goat-anti-mouse IgG (polyclonal, Southern Biotech) - Catalog # 1033-08.

Multiple lots were utilized for each of the antibodies listed above for the duration of the experiments presented in this manuscript. As such, providing all individual lot numbers is not possible. However, the most recent lot numbers are available upon request.

Validation information for R&D Systems antibodies: (https://www.rndsystems.com/products/rd-systems-approach-antibody-quality)
"R&D Systems carefully tests every antibody we produce to ensure outstanding performance. Our commitment to quality allows you to be confident in your results and help you generate publication-quality data. Details regarding the steps we take to provide you with only the highest-quality antibodies are listed below; Quality Control: Each antibody is manufactured under controlled conditions, undergoing rigorous quality control testing to ensure lot-to-lot consistency and outstanding performance in all applications listed on our datasheets; Endotoxin Level: Neutralizing antibodies are tested to ensure low endotoxin levels which are reported on individual datasheets. The formulation of most antibodies does not contain azide or other preservative; Extensive Specificity Testing: All antibodies are tested for cross-reactivity with closely related molecules using a variety of applications, including direct ELISA, to ensure specificity. These efforts are facilitated by our extensive library of in-house developed antigens; Formulation: For maximum stability, most antibodies are supplied lyophilized. This also facilitates shipping and storage."

Validation for Cell Signaling Antibodies: (https://www.cellsignal.com/about-us/cst-antibody-validation-principles)
"At Cell Signaling Technology (CST), we understand that there is no single assay that can determine the validity of an antibody. Confirming that an immunoreagent is sufficiently specific and sensitive depends on the application and protocol being used, the type and quality of sample being analyzed, and the inherent biophysical properties of the antibody itself.
To ensure our antibodies will work in your experiment, we adhere to the 'Hallmarks of Antibody Validation', six complementary strategies that can be used to determine the functionality, specificity, and sensitivity of an antibody in any given assay. CST adapted the work by Uhlen, et. al., ("A Proposal for Validation of Antibodies." Nature Methods (2016)) to build the Hallmarks of Antibody Validation, based on our decades of experience as an antibody manufacturer and our dedication to reproducible science."

Validation for Abcam immunoblot antibodies: (https://www.abcam.com/primary-antibodies/how-we-validate-our-antibodies)
"Antibodies are validated in western blot using lysates from cells or tissues that we have identified to express the protein of interest. Once we have determined the right lysates to use, western blots are run and the band size is checked for the expected molecular weight. We will always run several controls in the same western blot experiment, including positive lysate and negative lysate (if possible, Figure 2). When possible, we also include knock-out (KO) cell lines as a true negative control for our western blots. We are always increasing the number of KO-validated antibodies we provide. In addition, we run old stock alongside our new stock. If we know the old stock works well, this also acts as a suitable positive control. If the western blot result gives a clear clean band and we are happy with the result from the control lanes, these antibodies will be passed and added to the catalog."

Validation for BD Biosciences Antibodies: (https://www.bdbiosciences.com/en-us/products/reagents/flow-cytometry-reagents/research-reagents/quality-and-reproducibility)
"BD Biosciences identifies key targets of interest in scientific research and develops its own specific antibodies or collaborates with top research scientists around the world to license their antibodies. We then transform these antibodies into flow cytometry reagents by conjugating them to a broad portfolio of high-performing dyes." A world-class team of research scientists help ensure that these reagents work reliably and consistently. The specificity is confirmed using multiple methodologies that may include a combination of flow cytometry, immunofluorescense, immunohistochemistry, or western blot to test staining on a combination of primary cells, cell lines, or transfectant models. All flow cytometry reagents are titrated on the relevant positive or negative cells."

Validation for ThermoFisher Antibodies: (https://www.thermofisher.com/us/en/home/life-science/antibodies/invitrogen-antibody-validation.html)
"To help ensure superior antibody results, we've expanded our specificity testing methodology using a two-part approach for advanced verification. Part 1—Target specificity verification. This helps ensure the antibody will bind to the correct target. Our antibodies are being tested using at least one of the following methods to ensure proper functionality in researcher's experiments: Knockout—expression testing using CRISPR-Cas9 cell models; Knockdown—expression testing using RNAi to knockdown gene of

interest; Independent antibody verification (IAV)—measurement of target expression is performed using two differentially raised antibodies recognizing the same protein target; Cell treatment—detecting downstream events following cell treatment; Relative expression—using naturally occurring variable expression to confirm specificity; Neutralization—functional blocking of protein activity by antibody binding; Peptide array—using arrays to test reactivity against known protein modifications
SNAP-ChIP™—using SNAP-ChIP to test reactivity against known protein modifications; Immunoprecipitation-Mass Spectrometry (IP-MS)—testing using immunoprecipitation followed by mass spectrometry to identify antibody targets
Part 2—Functional application validation: These tests help ensure the antibody works in a particular application(s) of interest, which may include (but are not limited to): Western blotting, Flow cytometry, ChIP, Immunofluorescence imaging, Immunohistochemistry
Most antibodies were developed with specific applications in mind. Testing that an antibody generates acceptable results in a specific application is the second part of confirming antibody performance.

Validation for BioLegend antibodies: (https://www.biolegend.com/en-us/quality/product-development)
"BioLegend spends a considerable effort in developing and creating new reagents for research. We produce a variety of recombinant proteins, antibodies, immunoassays, and multiomics tools. The below example will focus on our stringency in creating new monoclonal antibodies produced from hybridomas:
Clones of these hybridomas are carefully selected based on a number of criteria including robust growth and efficient production of a single clone of antibody that is specific to the intended target. The best clones move on to applications testing. The steps include:
Immunogen design and construction, Immunization of host animal, Hybridoma creation, ELISA or application-specific screening of antibodies from clones, and Application testing, including WB, ELISA, ChIP, IF, IHC, or biofunctional assays.
Antibody clones are then tested in a variety of assays to see which applications they are suited for. As an example, clone 13A3-1 for phosphorylated STAT3 (Tyr705) demonstrated excellent performance in flow cytometry, western blot, and chromatin immunoprecipitation. Thus, the clone cross-validates itself by demonstrating functionality across orthogonal testing methods.
Additionally, the biological induction of the phosphorylated state using IL-6 further validates the specificity of the antibody."

Validation of Miltenyi Antibodies: (https://www.miltenyibiotec.com/US-en/products/macs-antibodies/antibody-validation.html#gref)
"With the introduction of recombinant antibodies in 2012, we made a significant investment into improving the quality and consistency of our antibodies. The standardized antibody production process, starting from a defined DNA sequence, and the nature of recombinant antibodies ensure high purity and lot-to-lot consistency.
In addition, recombinant antibodies do not display any undesired mixtures of heavy and light immunoglobulin chains, which is often the case with conventional hybridoma-derived antibodies[5] (PMID: 29485921). Furthermore, our REAfinity™ Recombinant Antibodies have a mutated Fc region that abolishes any binding to Fcγ receptors, resulting in a background-free analysis. These advantages make REAfinity Recombinant Antibodies ideal tools for improving experimental reproducibility.
Recombinant antibodies ensure high lot-to-lot consistency as compared to traditional hybridoma technology. Mass spectrometry analysis of the purified recombinant antibodies confirms the improved purity of antibody products.
During development of an antibody, a suitable test to verify specificity of the clone is performed. Several approaches are possible. Below you will find a list of methods we typically perform: Counterstaining - To validate the specificity of an antibody, a suitable counterstaining is performed, which verifies the target population; Target protein knockout - For this approach, the target gene is knocked out in a suitable cell line using site-specific nucleases and the knockout is confirmed by sequencing of the target locus.
The antibody is considered to bind specifically to the intended epitope, if no antibody binding to the knockout cells can be detected.
The antibody staining is controlled by fluorescence microscopy as well as flow cytometry; Epitope competition assay - In order to compare the epitope specificity of REAfinity Clones with other known clones in the market, competition assays are performed.
Cells are incubated with an excess of purified unconjugated REAfinity Antibody followed by staining with fluorochrome-conjugated antibodies of other known clones against the same marker."

Validation of Genscript Antibodies: (https://www.genscript.com/antibody-validation.html)
"Not all antibodies are valid for every experiment and condition, they must be validated for the specific application and species. Currently, there is no standard means of "antibody validation", and this can greatly impact experimental reproducibility and reliability. Journals and granting agencies have taken steps to address this gap. Many now have requirements to explicitly state how you will validate an antibody for a specific use. Unfortunately, there are no universally accepted criteria for antibody validation. So, it is up to each researcher to validate each and every antibody for the intended application.
Basic Validation:
Standard Antibody Validation Methods include western blot, ELISA, flow cytometry, and IHC. Most researchers are comfortable with these tried-and-true methods. The validation process can be time-consuming because the researcher or the manufacturer must validate the antibody for each application. This difficulty is compounded because each the conditions of each assay are different. For instance, a western blot depends on the denaturing of proteins. So, a western blot-validated antibody may work fine in denaturing conditions but may fail to recognize antigens in their native conformation (i.e., ELISA). Likewise, an antibody validated for native protein affinity could fail to bind the same antigen following denaturation or fixation.
The above methods play necessary roles in validating antibodies. However, we cannot rely solely on these methods, because they do not represent the ever-expanding applications. The confidence level of the validated antibodies can be increased by including other validation techniques."

Validation

All antibodies were purchased from commercial vendors, who provide validation information on their websites. We performed independent validation of Aiolos antibodies via flow cytometry and immunoblot analyses comparing wildtype and Aiolos knockout (Ikzf3-/-) samples .

Eukaryotic cell lines

Policy information about cell lines and Sex and Gender in Research

Cell line source(s)

MDCK cell line (BEI Resources, NIAID, NIH: Kidney (Canine), Working Cell Bank, NR-2628); Platinum-E (Plat-E) Retroviral Packaging Cell Line (Cat# RV-101, Cell Biolabs, Inc.).

Authentication	As MDCK cells and Plat-E cell lines were received from the Suppliers shortly before use, they have not been Authenticated further.
Mycoplasma contamination	As a new batch of MDCK cells were received from the supplier, this cell line was not tested for Mycoplasma contamination. During culture, Plat-E cells were tested weekly for Mycoplasma contamination, which was routinely negative.
Commonly misidentified lines (See ICLAC register)	*Name any commonly misidentified cell lines used in the study and provide a rationale for their use.*

Animals and other research organisms

Policy information about studies involving animals; ARRIVE guidelines recommended for reporting animal research, and Sex and Gender in Research

Laboratory animals	C57BL/6 mice were obtained from the Jackson Laboratory. Ikzf3-/- mice were originally obtained from Riken BRC and backcrossed to the C57BL/6 Jackson background for more than 10 generations to generate Ikzf3(-/-)/J mice. OT-II mice (with the transgene located on the y-chromosome, originally generated by the Carbone laboratory; Barnden et al. 1998, Immunol Cell Biol) were a generous gift of Dr. Haitao Wen. Ikzf3-/- mice were crossed to OT-II mice to generate OT-II Ikzf3-/- animals for adoptive transfer studies. Germline knockout studies involved the use of both male and female mice. As the OT-II transgene was located on the y-chromosome, adoptive transfer studies utilized only male mice (donors and recipients). For all in vitro studies, naive CD4+ T cells were isolated from 5-8 week old mice. For all influenza infection studies, mice were infected between 8 and 12 weeks of age. For each individual experiment and replicate, mice were age- and sex-matched.<br><br>Our standard mouse vivarium rooms are maintained at 72+/-4 degrees F, with a 12:12 light dark cycle, and humidity is maintained roughly between 30-70%. Animals are housed in individually ventilated cages (Allentown), are fed an irradiated natural ingredient chow diet ad libitum (Evnigo Teklad Diet 7912) and provided reverse osmosis purified water through an automatic rack watering system. Cages are bedded with ¼" corn cob bedding (Bed-o-Cobs, The Andersons) with a cotton square nestlet for nesting material. Animals are group housed whenever possible and cages are autoclaved prior to housing animals.
Wild animals	No wild animals were utilized in this study.
Reporting on sex	For both in vitro polarization and germline knockout influenza infection studies, both male and female mice (sex-matched between genotypes for each replicate and study) were utilized. For adoptive transfer studies, only male donor and recipient mice were used, as the OT-II animals utilized contained the TCR transgene on the y-chromosome.
Field-collected samples	No field-collected samples were utilized in this study.
Ethics oversight	All studies performed on mice were done in accordance with the Institutional Animal Care and Use Committee at the Ohio State University in Columbus, OH, which approved all protocols used in this study.

Note that full information on the approval of the study protocol must also be provided in the manuscript.

Flow Cytometry

Plots

Confirm that:

- [x] The axis labels state the marker and fluorochrome used (e.g. CD4-FITC).
- [x] The axis scales are clearly visible. Include numbers along axes only for bottom left plot of group (a 'group' is an analysis of identical markers).
- [x] All plots are contour plots with outliers or pseudocolor plots.
- [x] A numerical value for number of cells or percentage (with statistics) is provided.

Methodology

Sample preparation	Murine samples:<br>Mice were infected with influenza (A/PR8/34 or OVA323-339-expressing PR8) for 8 days. On day 8, whole blood was collected by cardiac puncture for serum antibody analyses. Draining lymph nodes were harvested and single cell suspensions were generated in tissue preparation media (IMDM + 4% FBS) by passing tissue through a nylon mesh strainer, followed by erythrocyte lysis via 3-minute incubation in 0.84% NH_4Cl. For lung single cell suspension generation, whole lungs were perfused with PBS and dissociated in Collagenase IV containing medium via Miltenyi Biotec GentleMACS Dissociator following the manufacturer's instructions. Dissociated tissue was then passed through a nylon mesh strainer and layered with Percoll. The mononuclear layer was harvested and erythrocyte lysis was performed as with DLN samples. For analysis of IL-2 production, homogenized samples were cultured in the presence of PMA and Ionomycin and protein transport inhibitors for 3h prior to staining. For analysis of granzyme B and perforin, homogenized samples were treated with protein transport inhibitors for 3h prior to analysis by flow cytometry. For adoptive transfer studies, whole-tissue homogenates were processed as above and stimulated with OVA323-339 peptide (AnaSpec) at 5ug/mL for 48h, with protein transport inhibitors added at 45h. Cells were washed 1X in ice cold FACS buffer (PBS + 4% FBS) before staining.<br>For analysis of antigen-specific CD4+ T cell populations in murine samples, cells were stained in FACS buffer with IAb NP311–

325 MHC class II tetramer (1:100, NIH Tetramer Core Facility) at room temperature for 1 hour. To address intra-replicate variability for the analysis of antigen-specific populations, the percentage of NP+ cells in Aiolos-deficient samples was presented relative to WT (a single WT sample was set as the control for each independent experiment, and each sample was normalized to this control).

For extracellular staining, samples were pre-incubated for >5 minutes at 4C with Fc Block (clone 93; BioLegend), then stained in the presence of Fc block for 30 minutes at 4C using the following antibodies: CD4 (1:300; clone GK1.5; R&D Systems); CD44 (1:300; clone IM7; BD Biosciences); CD62L (1:300; clone MEL-14; ThermoFisher); PD-1 (1:50; clone 29F.1A12; BioLegend); Cxcr5 (1:50; clone SPRCL5; ThermoFisher); CD25 (1:25; clone PC61.5; ThermoFisher); BD Biosciences), CD45.2 (1:100; clone 104; Biolegend), CD122 (1:50, clone 5H4; Biolegend), NKG2A/C/E (1:100; clone 20D5; Biolegend), and Ghost V510 or Red 780 viability dye (1:400-1:750; Tonbo Biosciences). Cells were then washed 2X with FACS buffer prior to intracellular staining. For intracellular staining, cells were fixed and permeabilized using the eBioscience Foxp3 transcription factor staining kit (ThermoFisher) for 30 minutes at room temperature, or overnight at 4C. Following fixation, samples were stained with the following antibodies in 1X eBiosciences permeabilization buffer for 30 minutes at room temperature: T-bet (1:100; clone 4B10; BioLegend); Foxp3 (1:300; clone FJK-16s; ThermoFisher); Bcl-6 (1:20; clone K112-9; BD Biosciences); IL-2 (1:50; clone JES6-5H4; BioLegend); Eomes (1:100; clone DAN11MAG; ThermoFisher); IFN-g (1:250; XMG1.2; BioLegend); Granzyme B (1:300; clone GB11; ThermoFisher); and Perforin (1:100; S16009A; BioLegend). For some experiments, samples were also stained with a panel of excluded (dump gate) antibodies: CD45R/B220 (1:100; clone RA3-6B2; Biolegend), CD11b (1:100; clone M1/70; Biolegend), F4/80 (1:100, clone BM8, Biolegend), and CD11c (1:100, clone N418, Biolegend). Cells were washed with 1X eBiosciences permeabilization buffer and resuspended in FACS buffer for analysis. Samples were run on a BD FACS Canto II or BD FACS Symphony and analyzed using FlowJo software (version 10.8.1).

Human samples:

Human tissues were collected and utilized in accordance with protocols approved by The Ohio State University Institutional Review Board. Donor consent was acquired when deemed appropriate according to the approved Ohio State University Institutional Review Board protocol. Human pediatric tonsils were obtained following overnight shipment after surgery via the CHTN Western Division at Vanderbilt University (Nashville, TN). Lymphocytes were enriched from fresh tonsil tissue specimens using previously reported protocols. Briefly, single-cell suspensions were generated by dissociation via a GentleMACS Dissociator (Miltenyi Biotech) according to the manufacturer's instructions. Cells were diluted in PBS (Thermo Fisher Scientific), layered over Ficoll-Paque PLUS (GE Healthcare), and centrifuged at 2000 rpm for 20 minutes at room temperature with the brake off and the mononuclear layers were harvested.

Lymphocyte populations were stained using methods similar to mouse sample processing using antibodies directed against surface or intracellular proteins according to the manufacturers' recommendations. Where appropriate, fluorescence minus one (FMO) controls were used to determine nonspecific staining. The LIVE/DEAD Fixable Aqua Dead Cell Stain Kit (Thermo Fisher Scientific) was used to exclude nonviable cells in the analysis. Intracellular staining was performed using the Transcription Factor/FOXP3 Fixation/Permeabilization Solution Kit (Thermo Fisher Scientific) according to the manufacturer's instructions. The following antibodies were used: CD44-APC (DB105, Miltenyi Biotec), CD4-AF700 (RPA-T4, Thermo Fisher Scientific), CD45RA-APC-Cy7 (REA1047, Miletnyi Biotec), AIOLOS-PE (16D9C97, BioLegend), CXCR5-PE-Vio770 (MU5UBEE, Thermo Fisher Scientific), FOXP3-PerCP-Vio770 (PCH101, Thermo Fisher Scientific), CD3-PE-Vio615 (UCHT1, BioLegend), CD8-FITC (REA734, Miltenyi Biotec), PD-1-BV421 (MIH4, BD Biosciences), CD62L-BV605 (DREG-56, BD Biosciences), CD45RO-BV650 (UCHTL1, Biolegend). Samples were run on a FACSAria II (BD Biosciences) and analyzed using FlowJo (BD Biosciences, version 10.8.1).

Instrument

mouse sample analyses: BD FACS Canto II ad BD FACS Symphony; human sample analyses: BD FACS Aria II

Software

Data collection: BD FACSDiva Software
Data analysis: FlowJo software (version 10.8.1)

Cell population abundance

As samples were acquired for analysis only, and no sorting was performed, population abundance in post-sort fractions is not applicable.

Gating strategy

Initial gating of lymphocyte populations (FSC vs. SSC) was based upon standard lymphocyte size and complexity. Further gating of populations was performed based either on comparison to unstained cellular control or isotype controls as appropriate.

☒ Tick this box to confirm that a figure exemplifying the gating strategy is provided in the Supplementary Information.
